# Tobacco industry thwarts ad ban legislation in India in the 1990s: Lessons for meeting FCTC obligations under Articles 13 and 5.3

**DOI:** 10.1016/j.addbeh.2022.107306

**Published:** 2022-03-14

**Authors:** Amit Yadav, Stanton A. Glantz

**Affiliations:** a Center for Tobacco Control Research and Education (CTCRE), University of California, San Francisco, USA; b The International Union Against Tuberculosis and Lung Disease (The Union), South East Asia Office, New Delhi, India; c Center for Tobacco Control Research and Education, Cardiovascular Research Institute, Division of Cardiology, Department of Medicine, University of California, San Francisco, CA, USA

**Keywords:** Advertising, Tobacco industry, Prevention, FCTC, India, Public policy

## Abstract

Bans on tobacco advertising are important for reducing tobacco-caused disease. Previously secret internal tobacco industry documents and organizational and newspaper websites related to tobacco control efforts in India during 1990s were analyzed. The Ministry of Health and Family Welfare, World Health Organization, Indian Council of Medical Research, and civil society played important roles in pushing for tobacco control legislation beginning in the 1980s. Guided by transnational tobacco companies, especially British American Tobacco, Philip Morris International, and RJ Reynolds, Indian cigarette companies formed the Tobacco Institute of India (TII). Following the industry’s global strategy, TII proposed voluntary advertising codes, used diplomatic channels and high level political and judicial lobbying, and allied with other industry, sports and trade groups to delay legislation for ten years. TII argued for the social and economic importance of tobacco and that laws were unnecessary, unconstitutional, and would hurt the economy. These early global strategies were continuing in 2022 to delay and evade legislative efforts to ban tobacco advertising. Understanding these strategies can inform public health efforts to counter industry efforts to thwart the WHO Framework Convention on Tobacco Control in 2022 not only in India, where the Ministry of Health and Family Welfare has proposed strengthening India’s tobacco control law, but globally.

## 1. Introduction

Tobacco companies use sophisticated advertising and marketing to promoting their products globally (World Health Organization, 2019). Worldwide efforts to rein in tobacco advertising started in the United Kingdom (UK) after the Royal College of Physicians’ 1962 report, *Smoking and Health* (Royal College of Physicians, 1962). In 1964, the tobacco industry responded with self-regulation, voluntary codes, freedom of expression defenses and youth smoking prevention programs to evade and undermine effective bans on tobacco advertising. The industry avoided legal restrictions until 2003 when the comprehensive UK Tobacco Advertising and Promotion Act was adopted (Harris et al., 2006; Hiilamo & Glantz, 2017; Mamudu et al., 2008b; Savell et al., 2014b; Sebrié & Glantz, 2007). The UK code prohibited advertisements targeting persons below age 21, advertising within 500 feet of elementary, junior and high schools; prohibited payments for using cigarettes as props in movies and limited sampling (Table S1). In 1984, the United States (US) tobacco companies established their first voluntary code (No author, 1990) and, as of 2021, had still evaded most legal restrictions on advertising and promotion. Both voluntary codes are based on the premise that smoking is an “adult choice” and nominally requires that tobacco advertising addresses only adult smokers (No author, 1990). However, the industry has a history of systematically violating these voluntary codes so they have no practical effect on reducing youth advertising exposure (Cox, 1984; Hastings & MacFadyen, 2000; Jennifer S Mindell, 1993; Richards et al., 1996; Robertson et al., 1998; Svati Chakravarty, 1998). Tobacco advertising continues to be a serious threat globally; the World Health Organization (WHO) reported that only 48 countries had passed complete tobacco advertising, promotion and sponsorship (TAPS) bans by 2018 (World Health Organization, 2019).

In 1990, relatively low smoking rates and growing population led the multinational tobacco companies to target India as a potentially profitable market (Barton, 1994) as part of their global expansion following trade liberalization. British American Tobacco’s (BAT) interest in India increased more than in any other Asian market (Group Planning Department, 1994) and Philip Morris International (PMI) also considered India a high potential market for its cigarette business (Philip Morris & Walk, 1997). BAT consolidated its position as India’s biggest tobacco company through Indian affiliate ITC Limited (established in 1910 as Imperial Tobacco Company of India Limited, renamed to India Tobacco Company Limited in 1970 and ITC Limited in 2001 (ITC Limited, 2020)). The tobacco companies considered avoiding advertising and marketing restrictions in India as crucial to their expansion (British American Tobacco Limited & Davis, 1994).

At the same time, following global tobacco control developments, Indian civil society called for strict regulation of tobacco, especially advertising. Studies by the Indian Council of Medical Research (ICMR) and others highlighted the role of tobacco advertising in youth uptake and suggested a legislative ban on advertising (Bhattacharjee et al., 1994; Gavarasana et al., 1991; Hamner et al., 1992). Encouraged by the WHO, civil society and health professionals in the 1990s, the Indian Ministry of Health and Family Welfare (MoHFW) proposed a comprehensive tobacco control law in 1994. The tobacco industry saw these rising voices as a serious threat to their future growth in India.

Acknowledging the serious threat posed by the proposed 1994 legislation, BAT, guided by its offices in London, in collaboration with PMI from the US and PMI’s Asia office in Hong Kong, facilitated creating the Tobacco Institute of India (TII) and helped TII implement the industry’s global strategies to delay restrictions on advertising (and other tobacco control policies) in India. Even though the MoHFW developed draft legislation in 1994 (Tobacco Institute of India & Sarkar, 1994), these industry tactics blocked legislation until 2003 when the Cigarettes and Other Tobacco Products Act (Ministry of Health and Family Welfare Government of India, 2003) (COTPA) passed. The tobacco industry understands that successful implementation of tobacco control policies in one country – especially a major country like India – would diffuse to other countries in the region and around the world (Crosbie et al., 2019; Hiilamo et al., 2014).

This paper details how the tobacco industry mobilized global resources to block tobacco control legislation during the 1990s in India. The experience from the 1990s remains relevant in India in 2022 as government and public health advocates in India work to strengthen COTPA based on amendments the MoHFW released for public comment (Ministry of Health and Family Welfare Government of India, 2021). India is also a hub for the South Asia Region for both the tobacco industry and tobacco control as they implement the WHO Framework Convention on Tobacco Control (FCTC).

## 2. Methods

We searched internal tobacco industry documents in the Truth Tobacco Industry Documents (TTID) (https://www.industrydocuments.ucsf.edu/tobacco/) between November 2019 and March 2020. TTID is the only comprehensive collection of previously secret internal tobacco industry documents available online. We started with the terms “Indian Government,” “Tobacco Products Bill 1994,” “Tobacco Institute of India,” “voluntary code” AND “Tobacco Institute of India,” “voluntary code AND India,” “British High Commission AND India” dated between January 1990 and December 1999. We conducted further searches using standard snowball techniques (Mamudu et al., 2008a; McDaniel et al., 2008) using the names of individuals, organizations and key events mentioned in the documents, examining adjacent documents (previous and next Bates) and the TTID’s “more like this” function. We retrieved 1,982 documents from the above search criteria and analyzed 103 documents that were related to tobacco industry efforts to block stronger tobacco control measures from 1990 to 1999, the period of interest in this paper (Table S2). Dr Yadav reviewed documents on the basis of their content related to advertising and marketing of tobacco products, industry-promoted voluntary codes and the proposed 1994 legislation. Prof Glantz verified the selected documents for their relevance and inclusion in the analysis.

We searched Google when full details of the individuals and documents were not available in the TTID to verify the names and affiliations of people (Table S3) and to source other documents and information not fully available at TTID.

## 3. Results

Table 1 presents a chronology of key tobacco industry efforts to block India’s tobacco control legislation in the 1990s and government and non-government actors to enact it.

### 3.1. Initial efforts to curb smoking and advertising

In 1990, the Tata Institute of Fundamental Research (TIFR) organized an international symposium on tobacco control in Mumbai (Hamner et al., 1992). Civil society groups, including consumer organizations, also mounted legal challenges to the tobacco industry in the 1990s that sensitized the government and judiciary to the need for comprehensive tobacco control legislation (Reddy & Gupta, 2004).

The Central Government directed its ministries and departments to ban smoking in health care establishments, educational institutions, conference rooms, domestic air flights, air-conditioned train cars and buses (No author, 1992). In 1991, the Ministry of Urban Development directed all municipalities to remove tobacco billboards, and the Health Minister told Parliament that a law was under consideration that would include stronger statutory health warnings on all cigarette packs and ban cigarette advertising (No author, 1992).

According to a document in the BAT collection, in July 1991, the MoHFW in collaboration with WHO and the All India Institute of Medical Sciences also organized the first National Conference on Tobacco OR Health (NCTOH), which recommended comprehensive tobacco control legislation including an advertising ban (No author, 1992).

### 3.2. Formation of tobacco Institute of India

Increased pressure for tobacco advertising and public smoking bans from MoHFW, WHO, health professionals and civil society prompted the three rival tobacco companies (ITC [a BAT subsidiary], Vazir Sultan Tobacco [VST, a BAT subsidiary]), and Godfrey Phillips India [GPI; a Philip Morris International subsidiary]) to work with their multinational parent companies to establish the Tobacco Institute of India to present a united political and public relations front (Tobacco Institute of India, 1992). TII was incorporated in November 1992 to lobby for “favorable treatment for the cigarette industry” (Sarker, 1992). TII’s new director Amit Sarkar, a former ITC employee, worked with Peter Clarke, BAT Company Secretary in London, to develop Articles of Association (ITC Limited & Sarkar, 1992; Sarker, 1992) and communications priorities and plans “to create awareness of and clarify issues related to the socioeconomic importance of tobacco in India, with particular regard to exports, agriculture and farmers, and government revenues” (Sarker, 1992).

TII responded to NCTOH’s advertising ban recommendation by asserting that tobacco was an adult product sold for three centuries and that advertising merely helped brand switching and ensured competition. Sharing the TII response (Table S6), the ITC chairman urged the Health Minister to follow the Japanese model of regulation (K. Chugh, 1992), which allowed market liberalization and tobacco marketing deregulation, including tobacco advertising on television. In Japan, these policies had contributed to increased smoking rates among teens and young adults, especially female, and an overall increase in the cigarette market (Lambert et al., 2004).

TII targeted a 1993 federal Cabinet decision and subsequent attempts by MoHFW to enact comprehensive tobacco control legislation. TII mounted a sustained lobbying campaign to promote the Indian tobacco industry’s self-regulation to policy makers, including Members of Parliament (Lutz et al., 2000; Maxwell et al., 2000), by presenting the tobacco industry global strategy of voluntary advertising codes to avoid strong legislation banning advertising (Chapman, 1980; J. S. Mindell, 1993). The multinational tobacco companies in Bangladesh, Sri Lanka, and Pakistan also used TII as a regional resource to implement industry strategies there (Philip Morris, 1996; Sarkar, 1992). BAT also shared Indian TII lobying points to opposed excise tax increases with its Kenyan representatives to oppose excise taxes there (British American Tobacco Limited & Opukah, 1995a).

### 3.3. MoHFW initiates comprehensive legislation

A 1993 ICMR report estimated that tobacco killed nearly one million Indians annually and found that India’s marked increase in smoking was mainly due to aggressive cigarette marketing (Indian Council of Medical Research, 1993). In response to this report and NCTOH recommendations, MoHFW drafted a comprehensive tobacco control law (Tobacco Institute of India & Sarkar, 1994). Unlike the Cigarettes Act of 1975 (Cigarettes Act 1975), which applied only to cigarettes, the proposed law applied to all tobacco products, including bidi, cigar, gutkha, and chewing tobacco. It also included provisions banning tobacco advertising and promotion, requiring smokefree public places, banning sales to minors and improving health warnings (Table S5). Although the proposed warnings were an improvement, they were weak by then-current international standards; by then some countries were already requiring graphic health warnings on the front of the cigarette packs (Hiilamo et al., 2014).

The Cabinet approved the proposed law in September 1993 and the Law Ministry in August 1994, and MoHFW prepared to introduce it in Parliament in late 1994 (British American Tobacco Limited, 1994).

### 3.4. Promoting a voluntary advertising code

Before the Health Ministry introduced its legislation in December 1994, the TII promoted its voluntary self-regulation advertising code (Table S1) consistent with its global strategy (Savell et al., 2014b; Ulucanlar et al., 2016). Steered by Shabanji Opukah, Manager of Consumer and Regulatory Affairs at BAT London, TII developed the code for India in consultation with the Vice President for External Relations from RJ Reynolds in the USA (Osmon, 1995) and the Corporate Affairs Director of Rothmans in the UK (Godfrey Philips India Limited & Poddar, 1996). TII received support from the international networks that BAT, PMI, RJ Reynolds and Rothmans created, especially the Tobacco Documentation Center (TDC), which gathered and disseminated information across countries for the multinational tobacco companies (McDaniel et al., 2008).

The tobacco industry often cultivates and creates allies like the local advertising associations to fight regulations and lobby the government on their behalf (e.g., US (Pollay, 1994), UK (Cronin, 2012), Europe (Neuman et al., 2002), Latin America (Sebrié et al., 2005; Sebrié & Glantz, 2007), and globally (Mamudu et al., 2008b; Savell et al., 2014b)). The International Advertising Association and Indian Advertising Association joined TII in opposing advertising ban legislation and organized a workshop to promote self-regulation (Tobacco Institute of India & Sarkar, 1996c). Despite saying they wanted to work with the Advertising Standards Council of India (ASCI), after the workshop (Tobacco Institute of India & Sarkar, 1996c) TII rejected ASCI’s proposal because it banned testimonials by celebrities, models holding cigarettes in their mouths and surrogate advertising (Shook Hardy & Bacon LLP, 1999). (Surrogate advertising uses brand imagery for one product for which advertising is restricted nominally to promote another product that is not subject to the restriction.) ASCI withdrew its tobacco industry voluntary advertising codes in December 1998 (Shook Hardy & Bacon LLP, 1999). We could not ascertain if TII followed its own voluntary code released in January 1996; later studies reporting tobacco industry violations of voluntary advertising codes refer to general ASCI advertising codes (Bansal et al., 2005; Svati Chakravarty, 1998).

### 3.5. Building internal constituency

A strategy of building an internal constituency with various industry trade groups (Savell et al., 2014b) was used to avoid introduction of a law banning advertising. In 1994, TII and its allies including the Indian Newspaper Society, World Federation of Advertisers, Indian Society of Advertisers and the Advertising Agencies Association of India wrote the Prime Minister, MoHFW and Ministry of Information and Broadcasting opposing the legislation (Sarkar, 1994). Editorials and opinions in newspapers repeating TII arguments appeared (Table S7) and were used by the TII to lobby policymakers (Sarkar, 1994).

### 3.6. International tobacco diplomacy

Norman Davis, the BAT London director responsible for India, approached the Economic and Commercial Counsellor at the British High Commission in India asking it to lobby the Indian Government against the proposed law and suggest voluntary codes (British American Tobacco Limited & Davis, 1994). The Counsellor urged Indian authorities to allow a voluntary agreement rather than a complete ban (British High Commission & Holmes, 1994). Although the TII continued lobbying and the tobacco control groups called for stronger legislation, Davis already knew in October 1994 that the legislation, supposed to be introduced in December 1994, was being postponed for a year (British American Tobacco Limited & Davis, 1994).

### 3.7. Using legislative and administrative processes to delay legislation

Responding to pressure for broader consultation on the proposed law, the Government referred the existing Cigarettes Act 1975 and its regulations for review by the Parliamentary Committee on Subordinate Legislation (Tobacco Institute of India, 1994) (COSL) to determine whether the 1975 legislation was consistent with Constitutional mandates (Parliament of India, 2020). TII tried to influence COSL through its and its allies’ submissions (No author, 1995b) (Table S4) that echoed the tobacco industry’s arguments (British American Tobacco Limited & Opukah, 1995c; No author, 1995a; Tobacco Institute of India & Sarkar, 1995). TII argued that advertising played a negligible role in youth smoking initiation and the cigarette companies do not advertise to recruit adolescents. TII asserted smoking was an adult activity and that the advertising was directed at adult smokers only. It further submitted that several countries with ad bans had seen increases in youth smoking and suggested that sports sponsorship by major manufacturers, including tobacco companies, is widespread and that the companies were entitled to be recognized for that sponsorship. BAT’s Opukah coordinated TII’s response, which was prepared jointly in London by BAT’s Corporate Affairs and Smoking Issues departments and approved by its Legal department (No author, 1995a).

Despite these industry efforts, health experts and civil society convinced COSL to support stronger legislation (No author, 1995b).

TII and its allies including the Tobacco Board of India (a Ministry of Commerce board that promotes tobacco cultivation and industry (Ministry of Commerce and Industry Department of Commerce, 1975)), trade unions, farmers and growers associations then lobbied for broader consultation demanding the MoHFW form an expert committee on the economics of tobacco use (Tobacco Institute of India, 1994). The MoHFW gave into the pressure and constituted the Expert Committee headed by the chairman of the Indian Institute of Public Administration and 11 other experts (Table S8) (Sarkar, 1995). The Committee would “undertake a comparative study on the economics of tobacco use inter-alia [among other things] examining the tax revenue and foreign exchange earnings, employment and consumer expenditure on the one hand and the cost of tertiary level medical care facilities for treatment of tobacco-related diseases, losses due to fire hazard, ecological damage due to deforestation and disposal of tobacco-related waste on the other hand with a view to making an economic study of the impact of tobacco consumption” and deliver its report 4 months later, in July 1995 (Sarkar, 1995).

TII, with support from BAT and PMI, slowed the proceedings (British American Tobacco Limited & Rudge, 1995; Tobacco Institute of India & Sarkar, 1996a, 1996d) by enlisting several members of the Indian parliament, the Tobacco Board of India, trade unions and other industry allies to lobby for a “balanced composition” of the committee (Table S8) (Sarkar; 1995). MoHFW subsequently reconstituted the committee in September 1996 and gave it another four months to submit its report (Tobacco Institute of India & Sarkar, 1996b). The first draft was not completed until October 1998 and the final report not completed until May 2000 (Tobacco Institute of India & Sarkar, 2000), nearly 5 years after the initial report was due. The report concluded that tobacco use was India’s biggest public health challenge (Reddy & Gupta, 2004).

### 3.8. Claiming the advertising ban is unconstitutional

The TII engaged retired Supreme Court and High Court judges to question the constitutionality of a tobacco advertising ban and in April 1996 distributed the opinions against ban to policy makers. ITC’s legal department sent a formal letter with a summary of the opinions to the Law Ministry (ITC Limited & Mehta, 1996).

### 3.9. The proposed national legislation is shelved

In early 1995, under pressure from five major trade unions, tobacco industry allies, the advertising industry, Members of Parliament, and the Tobacco Board of India and aware that national elections were scheduled in 1995, the government withdrew the legislation (Tobacco Institute of India, 1995a). The Prime Minister asked the Health and Labour Ministries to proceed slowly until a plan was devised to compensate tobacco growers and others for lost income (Tobacco Institute of India, 1995a). The election resulted in a fractured mandate with no clear majority for any party, causing constant changes in political dynamics with three prime ministers serving between 1996 and 1998. India faced additional general elections in February-March 1998 and again in September-October 1999. In 1996 H. D. Devegawda, who as the Chief Minister of Karnataka (a major tobacco growing state) had attended the international tobacco growers meeting in Bangalore and openly supported the industry, became prime minister (Tobacco Institute of India, 1995c).

In 1996, ITC paid £9 million for sponsorship rights for the men’s international cricket world cup tournament to be held in India to name the trophy “Wills Trophy” after its cigarette brand (Tobacco Institute of India, 1995a). The advertising industry feared that proposed legislation would not only jeopardize the deal to live telecast the tournament, but would also affect cigarette ads in stadiums. Succumbing to industry pressure, the government asked its agencies to assess the revenue loss from implementing the legislation (Tobacco Institute of India, 1995a).

Using tactics and strategies similar to the efforts against the 1994 proposed legislation, the industry delayed passage of updated legislation until 2003, when Parliament passed the Cigarettes and Other Tobacco Products Act with comprehensive restrictions on tobacco advertising building upon the provisions from the original 1994 proposed legislation. COPTA took effect in 2004, a decade after the 1994 legislation was proposed. Although COTPA incorporated FCTC Article 13 (marketing bans) it did not implement Article 5.3 (protection of the policy making process from industry interference).

## 4. Discussion

While India, like most countries, viewed its tobacco control legislation as a national matter, tobacco companies managed the political fight as part of a global strategy to prevent development of effective tobacco control policies everywhere by preventing individual countries from developing policies that could diffuse to other countries (Hiilamo et al., 2014; Ulucanlar et al., 2016). While the literature on tobacco industry interference in India concentrates on the post-COTPA period (Bhojani et al., 2013; A. Chugh et al., 2020; Sankaran et al., 2015; Yadav, Nazar, et al., 2018; Yadav, Singh, et al., 2018), this study shows key decisions and resources were developed during the 1990s by multinational parent companies which remain active in national regulatory measures.

As in the US (Richards et al., 1996), UK (Bradley, 1983; Cox, 1984; University of Bath, 2020b), Japan (Iida & Proctor, 2018), and elsewhere (Carter, 2003; McDaniel et al., 2008), cigarette companies formed national bodies like TII to ensure they had a coordinated voice. TII received support from the TDC and also communicated with the US Tobacco Institute for information. TII adopted the same strategies used by the tobacco industry in high (Carter, 2003; Givel & Glantz, 2001; Hiilamo & Glantz, 2013; Neuman et al., 2002) and low and middle income countries (Lee et al., 2012) as it lobbied to block effective control legislation by promoting weak voluntary codes (Tobacco Institute of India & Sarkar, 1996e) and undermine enforcement of tobacco-control regulations (Integral PR Private Limited, 1999). They also engaged third party allies, especially hospitality industry (Tobacco Institute of India & Chaudhery, 1997), advertising associations (Sarkar, 1994), tobacco growers (Tobacco Institute of India, 1995c), and trade unions (Tobacco Institute of India, 1995b) to influence public and policymaker opinions in India.

With the enactment of COTPA, which prohibited all tobacco advertising and promotion, the tobacco industry resorted to litigation (Yadav, Singh, et al., 2018) and engaged with filmmakers, actors, and celebrities (Bansal et al., 2005; Yadav & Glantz, 2021) to promote their products through brand and product placements in films (Goswami & Kashyap, 2006) and through surrogate advertising (Yadav et al., 2020). India has implemented regulations to counter the effect of tobacco imagery in films and television programs, notably requiring anti-tobacco advertisements before and at the intermission of films with smoking and a static health message on the screen when any tobacco imagery is displayed (Nazar et al., 2021; World Health Organization, 2015; Yadav & Glantz, 2021).

Voluntary advertising codes are the industry’s first line of defense against strong government regulation (Saloojee & Dagli, 2000) because tobacco companies do not meaningfully restrict advertising (Richards et al., 1996). During FCTC negotiations, the industry used similar strategies to propose international voluntary marketing standards as an alternative to the treaty through its Project Cerberus (Mamudu et al., 2008b).

Using overseas diplomatic offices to lobby for the tobacco industry the way BAT used the British High Commission in India continued elsewhere. In March 2015, the British High Commissioner in Pakistan lobbied with BAT to persuade the Pakistani government to reverse health warning legislation (Kmietowicz, 2015). In August 2019, a Swiss diplomat lobbied Moldavian authorities against tobacco legislation for PMI (University of Bath, 2020a). Similar high level lobbying was continuing in Asia (Hopkinson et al., 2015), Africa (Patel et al., 2007), and elsewhere (Savell et al., 2014a). However, in 2013, the UK Department of Health and the UK Foreign and Commonwealth Office issued guidelines for overseas posts consistent with the FCTC, especially Article 5.3, and banned its diplomats from supporting the tobacco industry and interfering with national tobacco control efforts (Department of Health UK and Foreign and Commonwealth Office UK, 2013).

TII was still active as of 2021 (Tobacco Institute of India, 2021c), when it continued to engage the media through press releases (Tobacco Institute of India, 2021a) and oppose FCTC implementation by claiming that it is not mandatory for the Indian government and that FCTC Article 5.3 was arbitrary and undemocratic (Tobacco Institute of India, 2021b). In particular, on January 1, 2021, the MoHFW released a draft bill with proposed amendments to COTPA for public comment. The proposed Amendment Bill ended designated smoking area/rooms, removed concessions on point of sale advertising, increased the legal age of access to tobacco from 18 to 21 years, ban sale of loose cigarettes, introduced vendor licensing and provisions to comply with the FCTC Protocol to Eliminate Illicit Trade in Tobacco Products, and increased the penalties for violations (Tobacco Control Department et al., 2021) (Table S9). Shortly thereafter major voices opposing the bill started coming from the tobacco industry allies, including farmers, restaurant and retailers’ associations, while the TII called the amendments draconian (Writankar Mukherjee & Bureau, 2021).

Because the global tobacco industry used India as the gateway for access to south Asian markets and developed TII as a regional resource center, the lessons from India serve as cautions for governments in the region to protect their tobacco control policies from the tobacco industry and its front groups. Considering that BAT, PMI and others, including the US Tobacco Institute, were advising TII, the main arguments against the tobacco control efforts and approaches of the TII remained similar to the global experiences documented earlier. TII interference also remained similar over time, from blocking the 1994 legislation to delaying the 2003 COTPA legislation and further interference with the proposed COTPA amendments in 2015 and 2020. In 2016–17, 6 million farmers were growing tobacco on 0.45 million hectares land and there were still 267 million tobacco users in India. Even though these numbers represent only 5% of farmers (Agarwal, 2021) and 0.3% of land under cultivation (Vikaspedia, 2021), the TII successfully used tobacco farmers, and retailers to delay, dilute, and derail tobacco control measures. It also tried to use smokers to oppose stronger smoke free public places laws in 2008. However, the petition by a smoker along with others was set aside by the Supreme Court of India, paving the way for implementation of the rules (Supreme Court of India, 2008).

Similar to its strategy elsewhere (Ulucanlar et al., 2016), including Nepal (Bhatta et al., 2020), the tobacco industry worked in India not only through hired consultants or parliamentary and expert committees, but also approached retired supreme court and high court judges to argue that the proposed ban on tobacco advertising was unconstitutional, unnecessary and would have unfavourable social and economic consequences. These strategies (creating a national manufacturers’ association, proposing voluntary advertising codes, using allies, trade groups and media, international diplomacy and high level lobbying) were similar to those used by the industry to block tobacco control legislation in Brazil (British American Tobacco Limited & Opukah, 1995b), Nigeria (Egbe et al., 2017) and other LMICs (Lee et al., 2012; World Health Organization, 2000).

### 4.1. Policy implications and lessons

The Indian experience in the 1990s warns all countries that the tobacco industry continues to deploy similar strategies to prevent implementation of the FCTC. Although FCTC Article 13 (World Health Organization, 2004) and its guidelines (World Health Organization, 2008a) mandate a complete prohibition on tobacco advertising and those principles are reflected in COTPA, the industry has continued to circumvent these provisions through point of sale, surrogate advertising and promotions in films, TV and live streaming (Arora et al., 2012; Arora et al., 2021; Chaudhry et al., 2007; Goel et al., 2014; Paul, 2019; Sushma & Sharang, 2005).

FCTC Article 13 guidelines (WHO Framework Convention on Tobacco Control Conference of Parties, 2008) consider Corporate Social Responsibility (CSR) by tobacco companies tobacco advertising that should be banned under advertising and promotion provisions of national tobacco control laws. Nevertheless, Indian tobacco companies continue to continue CSR activities because the Corporate Act 2013 every company to spend 2% of its profit on CSR activity (Ministry of Corporate Affairs, 2013). Public interest litigation in the Chennai Hight Court led to a 2016 order to the Corporate Affairs Ministry requiring that any such CSR must comply with COTPA, which prohibits tobacco company advertising or promotion of their products or brands, including in their CSR activities (Ministry of Corporate Affairs, 2016). Indian tobacco companies have worked around COPTA by using their corporate logos (which are also used on branded products), brand extensions, brand sharing and corporate identity (name, logo and tag lines of the tobacco company) and by making high profile donations to funds (including COVID-19 relief) for the prime minister and provincial ministers (Yadav et al., 2021). One solution to this problem would be replace the existing CSR requirement with a 2% tax on tobacco company profits and for the government to distribute the funds to meet social needs, including tobacco control (Yadav et al., 2021).

Governments should avoid undisclosed interactions with the tobacco industry and follow a comprehensive code of conduct requiring disclosing interactions (World Health Organization, 2008b). Whereas COPTA implements FCTC Article 13, there is no provision equivalent to Article 5.3 (protection against industry interference). The recurring tobacco industry tactics in India and other countries necessitate the adoption of legislative, judicial and administrative measures to implement Article 5.3 mandates. As of September 2021, only the MoHFW had adopted a code of conduct for itself and all of its departments, organizations, institutions and agents (Tobacco Control Division & Ministry of Health and Family Welfare, 2020) despite a 2010 directive from the Karnataka High Court (Rao et al., 2016) to do so, allowing the tobacco industry to continue to interfere with tobacco control policy making and implementation in India through other ministries and departments (Bhojani et al., 2013; Yadav et al., 2020). Organizations like the Tobacco Board of India and the Central Tobacco Research Institute and the TII continue to lobby and support the tobacco industry and interact with policy makers with no conflict of interest declaration requirements or other preventive measure stipulated under Article 5.3 and its guidelines (A. Chugh et al., 2020).

Just as nongovernmental organizations and the World Health Organization played an important role in initiating efforts to enact tobacco control legislation in the 1990s, these players – together with supportive international networks – remain important in India and elsewhere (Bhatta, Bialous, et al., 2019; Bhatta, Crosbie, et al., 2019; Bhatta et al., 2020; Crosbie et al., 2016, 2017, 2018; Hiilamo et al., 2014; Uang et al., 2017, 2018), both in passing and supporting the implementation of tobacco control legislation, including in low and middle income countries. These connections need to continue to be supported and expanded.

### 4.2. Limitations

This paper focuses on developments in India during 1990–1999 as documented in the tobacco industry internal documents. Our analysis may not cover all the strategies and counter strategies which are not available in the TTID including complete information related to the tobacco control bill proposed in 1994 or the overall response from the tobacco industry or the other stakeholders. We only used English-language publications. Some of the developments described are based on information in the tobacco industry documents that could not be independently verified from other sources.

## 5. Conclusion

In the 1990s, BAT, PMI and other multinational tobacco companies applied global strategies to protect their Indian cigarette subsidiaries. These early global strategies continue to form the core of industry strategies to delay and evade legislative efforts to ban tobacco advertising in India. Tobacco companies successfully stymied the 1994 legislation. India did not get its national tobacco control law until 2003 effective in 2004, a decade later. In the same way the proposed amendment to COTPA in 2015 was withdrawn due to industry pressure. As of September 2021, it had been nearly a year since COTPA Amendment Bill 2020 was released with no further progress. It had not even been introduced in Parliament, much less adopted or implemented. Understanding these industry strategies can inform public health efforts to counter industry efforts to thwart the proposed COTPA amendment in India and implementation of the FCTC globally. In particular, government and law makers should be aware of the vested and commercial interests of the tobacco industry and the fact that TII is constituted as a lobby group of, for and by the tobacco industry. Since its establishment, TII has been working to advance tobacco industry interest by engaging with policy makers both directly and indirectly by putting forth and supporting front groups. Farmer and retailer groups as well as hospitality association and trade unions remain the most plausible carriers of tobacco industry narratives on behalf of the tobacco industry. Government and institutions need to be aware of these commercial and vested interests of the tobacco industry and their front groups and ensure that such interests do not get included or engaged in the policy making and policy implementation processes. The TII continues to be an industry lobby and resource for India and the region, highlighting the need for greater national and regional cooperation and collaboration among civil society networks and governments in the region to counter tobacco industry efforts and protect national tobacco control activities.

## Supplementary Material

supplement

## Figures and Tables

**Table 1:** Tobacco industry tactics to block the national law on tobacco control in India from 1990 to 1999.

Date	Government/NonGovernment and Tobacco Industry Organizations involved	Agencies/institutions approached or targeted	Tobacco industry activities / arguments	Tobacco control activities involved/challenged or undertaken by various actors
Jan 90	TIFR	MoHFW, WHO, NGOs		Organized international symposium on tobacco control.
May 90	Government of India	All federal ministries and departments		Ban on smoking in certain public places
Jul 91	MoHFW	WHO, ICMR, Tobacco industry, NGOs	Vehemently opposed all the recommendations except public education	1st NCTOH
May 92	TII	BAT	Share the details of formation of TII	
Dec 92	ITC	Health Minister	Oppose the recommendations of 1st NCTOH and proposed that the government should have dialogue with the Industry and TII.	
Sep 93	GPI	TII	Questions ICMR study on ‘Role of Health Personnel in Tobacco Control’ published in May-June 1993.	ICMR: increase in smoking due to ads.
Sep 93	BAT	TII	Suggests that ICMR study makes sweeping claims without citing evidence to support them.	Cabinet approves comprehensive law banning all forms of advertising*
Oct 93	TII	Media	Organized two-day media seminar to promote industry’s positions including on tobacco advertising.	
Mar 94	TII	Policymakers, bureaucrats and legislators	Lobby against ad ban of a legal product. Claims no correlation between juvenile smoking and advertising.	
May 94	TII	Policymakers, bureaucrats and legislators	Ad ban would harm the industry and tobacco farmers. Permit industry self-regulation.	
May 94	Tobacco Board of India	Ministry of Commerce	Keep the proposed law in abeyance and appoint expert committee for wider discussion.	
Jun 94	MP	Prime Minister	35 MPs demand review of the legislation and wider consultation with farmers and industry.	
Aug 94	BAT	British High Commission, India	Legislation would severely restrict marketing and have a serious impact on BAT’s business interests in India.	
Nov 94	All India Bidi, Cigar and Tobacco Workers Federation	Media	Threatened to launch an agitation against the legislation. Appoint an expert committee for wider consultation.	
Feb 95	Various Trade Unions	Prime Minster, Health Minister and Labour Minister	Submit representation against the legislation requesting consultations with tobacco growers’ and trade unions.	
May 95	TII and other allies (Table S4)	Parliamentary Committee on Subordinate Legislation	No role of advertising in initiation of smoking by young people. Advertising is only directed at adult smokers.	MoHFW, WHO and NGOs (Table S4) support the legislation.
Sep 95	ITC and TII	MoHFW	Inadequate representation of economists on the Expert Committee. Demand removal of tobacco control experts.	NGO Green Motherland advocated for smoke free public places
Jan 96	TII	Policymakers, bureaucrats and legislators	Voluntary code best way to regulated cigarette ads.	
Apr 96	ITC	Law Minister	Lobby against ad ban citing the Canadian and Indian Supreme Court decisions and other legal opinions.	
Apr 96	TII	Policymakers, bureaucrats and legislators	Publish legal opinions against the ad ban law. Voluntary code instead of a law is in the interest of the consumer.	
Jul 96	IAA, TII, ASCI	Various stakeholders and media	Support selfregulation for tobacco advertising. ASCI to help the tobacco industry devise a voluntary code.	Civil society calls for complete ban on tobacco advertising.
Oct 96	Members of Parliament	Health Minister	Industry biggest supporter of sports in India. Expert Committee’s conclusions will have adverse effect.	Civil society files litigations against sponsorship of sports by tobacco companies
May 99	HRIDAY	Prime Minister		Petitioned comprehensive ban on all tobacco advertising
Jan 99	BAT, TII	Expert Committee on Economics of Tobacco use in India	Report overlooks clear primary health care inadequacies. Moralizing “nanny state” at its worst.	MoHFW, health experts and NGOs support stronger legislation.

ASCI-Advertising Standards Council of India; BAT-British American Tobacco; GPI-Godfrey Phillips India Limited; IAA-Indian Advertising Association; ITC-ITC Limited; MoHFW-Ministry of Health and Family Welfare; MP-Member of Parliament; NCTOH-National Conference on Tobacco OR Health; NGO-Non Government Organization; TIFR-Tata Institute of Fundamental Research; TII-Tobacco Institute of India; WHO-World Health Organization;

* The law proposed to: Ban sale of tobacco products within 100 m of educational institutions and hospitals, ban smoking in public places, complete ban on all forms of advertising and prescribe prominent and effective health warnings.

## Data Availability

All data are publicly available at the cited sources.

## Abbreviations:

ASCI: Advertising Standards Council of India
BAT: British American Tobacco
COPTA: Cigarettes and Other Tobacco Products Act
COSL: Parlimentary Committee on Subordinate Legislation
CSR: Corporate social responsibility
FCTC: WHO Framework Convention on Tobacco Control
GPI: Godfrey Phillips India
IAA: Indian Advertising Association
ICMR: Indian Council of Medical Research
ITC: India Tobacco Company Limited, later ITC Limited
MoHFW: Ministry of Health and Family Welfare
MP: Member of Parliament
NCTOH: National Conference on Tobacco or Health
PMI: Philip Morris International
TCD: Tobacco Documentation Center
TIFR: Tata Institute of Fundamental Research
TII: Tobacco Institute of India
TTID: Truth Tobacco Industry Documents Library
UK: United Kingdom
USA: United States of America
VST: Vazir Sultan Tobacco
WHO: World Health Organization
