# Supplementary material for: Tobacco industry thwarts ad ban legislation in India in the 1990s: Lessons for meeting FCTC obligations under Articles 13 and 5.3
Source: Addict Behav. Author manuscript; Available in PMC 2023 Jul 1. (PMC9942803; doi:10.1016/j.addbeh.2022.107306)
Supplement: supplement [file NIHMS1873704-supplement-supplement.docx]

**TOBACCO INDUSTRY THWARTS AD BAN LEGISLATION IN INDIA IN THE 1990s: LESSONS FOR MEETING FCTC OBLIGATIONS UNDER ARTICLES 13 AND 5.3**

**Amit Yadav and Stanton A. Glantz**

**Online Supplement**

| **Table S1: Voluntary advertising codes proposed by the Tobacco Institute of India** | | | | | | |
| --- | --- | --- | --- | --- | --- | --- |
| **Voluntary code in UK as in 1990** (Unknown, 1990) | **Proposed by TII on 25 Feb 94** (Unknown, 1994b) | **Modified by TII on 18 Apr 94** (Unknown, 1994c) | **Input from Rothmans, UK (on TII draft code)** (Godfrey Philips India Limited & Poddar, 1996) | **Released by TII in Jan 96** (Tobacco Institute of India, 1996) | **Inputs from BAT (on TII draft code)** (British American Tobacco Limited & Opukah, 1996) | **Advertising Standard Council of India** (Shook Hardy & Bacon LLP, 1999) |
| Not target person below the age of 21 years and within 500 feet of elementary, junior and high schools, | Purpose of the code is to establish self-regulation of cigarette advertising and promotion by members of TII | Observing standards of ethics since 1975, this code shall be the basis for tobacco products advertising in India | Add: Cigarettes are a legal product, and manufacturers have the right to inform their customers about their products. | Added following definitions in purpose and scope   - Advertising: activities conducted for brand awareness using the four mass media (TV, radio, newspapers and magazines) as well as Point of Sale material and Bill Boards. - Sales promotion: activities other than advertising activities e.g. benefits given to consumers and retailers | Agree with Bevan’s suggestion | Not to target minors or nonsmokers |
| No payment for placement of products in movies for general public | Cigarette advertising shall be directed to adults only to effect brand change and not directed to person below 18 years | Advertising directed only to adults and not to person below 18 years. No person below 25 to be depicted in ads, no health claims, not imply that all smokers, or essential to success, public prominence or romance or exaggerated satisfaction | Definition 'Advertising' should include Point of Sale material and billboards. | Statutory warnings to be displayed on packets and printed ads of tobacco products as required under the Cigarettes Act 1975 |  | Not depict more than one-third of a group of persons smoking |
| No one appearing under 25 years to be depicted in cigarette advertising | Restriction on Media:  No ad on state run TV or radio or cinema with rating or publications directed to minors or video cassettes for public or private showing | Advertised on state-run TV or radio only during adult viewership hours as may be specified from time to time. No ads on cinema screen for film rated for children, publications for minors and billboards placed less than 50 meters from school, hospital and place of worship. | Add: "No tobacco advertisement shall involve politics or religion". | Tobacco advertising shall not be directed at minors i.e. those below the voting age. Advertising only to brand switch and brand loyalty. No person below 25 to be depicted in ads, no health claims, not imply that all tobacco users, not involve politics and religion | Agree with Bevan’s suggestion | Not showing cigarettes in models' mouths |
| Not to imply that smoking is essential to social prominence, sexual attraction, attractiveness, good health | Ads shall not:  Depict person under 25 years; claim health properties; imply that all persons in the ad are smokers or smoking is essential to success; public prominence and romance; show exaggerated satisfaction | Cigarette sales promotions, gift or premium merchandise to be directed at adults and not minors |  | No ads on state run TV and radio except as agreed from time to time between parties concerned, not in cinema or publications meant for children and billboard placed within a reasonable distance of elementary or high school. |  | Not employing models younger than age 25 |
| Not depict a well-known athlete or show physical activity beyond that of normal recreation and no celebrity testimonials or other who appeal persons below 21 years of age | Statutory warnings to be displayed as per law on cigarette packs and printed and illuminated ads except on small portable objects, decoratives for personal, domestic or business use or object worn | Direct sampling limited to adults. Sampler shall ask for age of proof from person not clearly over age of 18 years. Limited to those willing to accept sample. Undertaken only by adult. |  | Sales promotion not directed at those below voting age. Only target over voting age | Instead of “tobacco users” just refer to “people above the voting age” | Not to imply that smoking is safe, healthy or popular, indicates manliness or emancipation, is attractive to the opposite sex, is associated with success, or facilitates concentration or relaxation |
| No samples: to under 21 years, at public places, in a vehicle, near educational institutions, mails or through telephone request. | Outdoor advertising not to be placed within 150 meters from school, hospital or place of religious worship. | sponsor events or functions which are not directed to minors, use only company name brand and trademark. No depiction of cigarettes or persons smoking |  | Direct sampling of tobacco products limited to those above voting age. Proof of age for not clearly above voting age. Vending machine permitted only if manned by a person above the voting age to prevent access to person below voting age | Delete reference to “tobacco users” | Not using testimonials by celebrities or role models |
| Samples only intended for smokers. | Cigarette sales promotions, gift or premium merchandise to be directed at adults and not minors | Members shall respect the marketing activities, materials and property of all  other members |  | Sponsorship of any event shall not direct as those below voting age |  | Not to appear in mediums intended for minors |
| Manufacturers and contractors to follow the sampling code | Direct sampling of cigarettes only to adults. Proof of age from those who appear minor. No minor or youth to sell or promote cigarettes | All employees, agents contractors to follow this code. Any concern regarding conformity to be submitted to the TII in writing |  | Members shall respect the marketing activities, materials and property of all  other members | Define “members” whether “members of TII” or “all people and organizations engaged in the tobacco trade” | No surrogate advertising |
| No mail distribution of nontobacco premium items bearing cigarette brand, logo etc. without written signed consent that such person is 21 years or older, a smoker. | May sponsor events or functions but not directed to minors, use only company name brand and trademark. No depiction of cigarettes or persons smoking | Code binding on all members. Members to give six months notice to withdraw adherence with the code to TII. Code may be amended from time to time. |  | All employees, agents contractors to follow this code. Any concern regarding conformity to be submitted to the TII in writing. TII to monitor compliance directly or through third party. |  | Not providing certain incentives for increasing tobacco sales |
| No other distribution of nontobacco premium items except with the purchase of a package or carton or to a person 21 years or older | Members shall respect the marketing activities, materials and property of all  other members |  |  | Code binding on all members. Members to give six months’ notice to withdraw adherence with the code to TII.  Code to apply to other segments of the industry.  Code may be amended from time to time | Delete last para in point 10.1 (code to apply to other segment of the industry) |  |
| Clothing bearing cigarette brand or logo to be only in adult size. | Self-regulation: All employees, agents contractors to follow this code. Any concern regarding conformity to be submitted to the TII in writing |  |  |  |  |  |
|  | Code binding on all members. Members to give six months notice to withdraw adherence with the code to TII. Code may be amended from time to time. |  |  |  |  |  |

| **Table S2: Tobacco Industry Documents related to tobacco industry interference in blocking the tobacco control legislation in India (1990-1999)** | | | | | |
| --- | --- | --- | --- | --- | --- |
| **Date** | **Event/Activity/**  **Order/**  **Communication** | **Organized/issued by** | **Targeted at** | **Key message** | **Tobacco Document Reference*** |
| Jan-91 | Direction to control advertisement of cigarettes and other tobacco products through municipal corporations | R.R.S. Pawar Director Local Self Government, Ministry of Urban Development, Government of India | All local self-government bodies | Remove cigarette advertising billboards | rkkk0203 |
| Apr-91 | Parliamentary question on proposed law | B.K.Hariprasad and shamim Hashmi | Ministry of Health and Family Welfare | Q: By when Central Government propose to introduce Bill containing comprehensive anti-smoking measures? A: Measures have been taken to control tobacco including prohibition on smoking in public places. Proposed law is under examination in consultation with Ministry of Law and other concerned authorities. Main feature include:  Statutory warning displayed on all cigarette packs e.g. smoking can lead to oral cancer, smoking can cause heart problems, smoking may reduce your life span, smoking can aggravate respiratory problem. Ban on advertisements of cigarettes. | rkkk0203 |
| Jul-91 | National Conference on Tobacco Or Health | Ministry of Health and Family Welfare World Health Organization | Government policy makers, legislators, tobacco industry, civil society, research institutions | The goal of tobacco-free society is not negotiable. An integrated educational, legislative and agro-economic strategy with an operational framework and political, administrative, financial and research support, is needed to protect other people from the tobacco menace and move in the direction of a tobacco free society | rkkk0203 |
| Mar-92 | Formation of Tobacco Institute of India | ITC Ltd., GPI ltd., VST Ltd. | Tobacco industry, Government of India | Article of Association of the Tobacco Institute of India circulated to the three sponsoring companies for approval. | fpkk0203 |
| Apr-92 | Tobacco and Health Scenario in India | Amit Sarkar, BAT | R A Mazumdar, Bangladesh V. Malalasekera, Sri Lanka NS Hameed, Pakistan | Shared the developments in India and the recommendations of the NCTOH and the strategy undertaken by the Indian tobacco industry to counter tobacco control strategies. | rxhh0214 |
| May-92 | Formation of TII | Amit Sarkar | Peter Clarke BAT | Details of the formation of TII along with a copy of its draft memorandum and articles of association shared for comments and advice. | yykk0203 |
| Jun-92 | Formation of Public Affairs Resource Group (PARG) | David Bacon | BAT globally | To achieve competitive advantage in public issues management with better communication and resourcing of public affairs plans and activities. | hhfw0200 |
| Dec-92 | Tobacco policy and the example of Japan. In response to the recommendations of the NCTOH | K.L. Chugh, Chairman ITC Ltd. | M.L. Fotedar, Union Minister for Health | Government policy is to develop programme after dialogue with Industry. Tobacco Institute of India represents the tobacco industry interests. We should follow example of Japan. Statutory warnings on all packs but advertisements allowed in all media No relation between smoking and health Major tobacco use in India is non-cigarettes Tobacco industry is source of biggest foreign exchange and help to bridge budget deficit. Government should focus on food and nutrition of our population, potable drinking water, basic sanitation, infant mortality, blindness, population control and HIV/AIDS. Any diversion from these will be a tragic waste of national resources. | sxhh0214 |
| Dec-92 | Formation of TII | Amit Sarkar | P Clarke, BAT | TII was incorporated on 25th November 1992 as a guarantee company by ITC Ltd, VST and GPI. Its main objectives are to promote, protect and safeguard the interest of the tobacco industry, particularly those of the cigarette manufacturers in India. The TII has already begun communications with minsters, bureaucrats, media as well as those in Industry and the ITC group to clarify the socio-economic importance of tobacco in India especially for farmers, exports, agriculture and govt revenues. Sharing two of the seven booklets to be used. | sfnd0196 |
| Jan-93 | Regional PARG Conference | Tobacco Institute of India | David Bacon | Thank you for giving opportunity to host BAT’s Regional PARG Conference and smoking issues/media training workshop. | hhgw0201 fhgw0201 |
| Sep-93 | Proposed legislation to regulate all tobacco products | The Union Cabinet | Ministry of Health and Family Welfare/Tobacco industry | Approves the Ministry of Health and Family Welfare proposed legislation. | ypvp0201 |
| Feb-94 | Letter to Editor | Dr Sharad Vaidya | Economic Times | The TII even before its formation through its constituents, stifled the proposal to ban all tobacco advertisements in the country in 1986. | tfwf0194 |
| Feb-94 | Voluntary Code Draft for discussion produced by TII | TII | Tobacco industry  Policy makers | TII releases a draft voluntary code for the marketing of cigarettes in India. | xfvw0110 |
| Mar-94 | TII newsletter | Tobacco Institute of India | Tobacco industry  Policy makers | We should not ban advertising of something which is legal to buy. Smokers have lowest cancer and heart disease rates in Australia. Beneficial effects of tobacco consumption Smoke to be healthy | ggwf0194 |
| Mar-94 | Industry set to fight sweeping ad ban | Industry press monitoring | Tobacco industry | Opposing the law, Amit Sarkar of TII was quoted as saying, “There is no need to ban tobacco advertising when such a ban does not exist even in USA, Britain, Germany, Japan, China or Russia.” | qhxx0210 |
| Apr-94 | BAT industries future business environment – BAT sees India as key market. | Group Planning Department, BAT | BAT | Within the total population of 890m, India has a huge middle class estimated at 225m, which is nearly the size of the entire US population, and growing at 20m p .a There are 280 adult males (most smokers are men), of whom 75m are affluent. This number could grow to 150m affluent males by the end of the century. 70% of the population are urban Most smokers still smoke Bidis which are about one sixth the price of cigarettes. Some of the difference is due to taxation which is levied at a much higher level on cigarettes and is much more likely to be paid than the tax on Bidis. The Tobacco Institute of India was formed in 1992 to lobby for favorable treatment for the cigarette industry. If all tobacco consumed in India was in the form of cigarettes, India would be a 450bn sticks per annum market. | flpp0136 |
| May-94 | WHO Award | World Health Organization | Tushar Kanti Das, Joint Secretary, Ministry of Health and Family Welfare | WHO Award for outstanding work in the field of Tobacco and Health. | ltdf0194 |
| May-94 | Indian Cancer Congress | Chris Proctor | Amit Sarkar | If you believe that the Congress is likely to produce significant adverse reaction and media attention, or provide a forum for anti-smokers, then there are several steps that could be taken.  Linda Rudge and I can monitor and report to TII on events that could cause problems, help prepare alerts or press releases. It will be helpful to have access to one of the speakers at the conference. Dr Tony Springall is due to give a presentation. He would be prepared to make statements to media. It would be extremely helpful to have an Indian specialist attend the meeting and be prepared to make Comment on the proceeding. | kfwf0194 |
| May-94 | TII Newsletter | TII | Tobacco industry Policymakers | Reproduces article by Khuswant Singh “Dhumrapan Nishedh” published in Hindustan Times on May 14, 1994. Wherein he concludes the ban on cigarette advertising will do great harm to the industry and to tobacco farmers. TII says, if such a legislation is enacted, it could open the floodgates to a “nanny state” approach which if permitted, would inevitably extend to a host of other products. A ban on advertising will make it impossible “to help tobacco growers” as envisaged by the Finance Minister. Government should reconsider and evolve a process of self-regulations by industry which would be infinitely more effective. | qfwf0194 |
| Jun-94 | Proposed Legislation/Indian Cancer Congress | Chris Proctor writes to Amit Sarkar requesting for a copy of the proposed legislation after seeing BMJ article on the proposed law. | Amit Sarkar Tobacco Institute of India | It is possible that some of the presentations at the cancer congress and some of the ensuing media will be seen as supportive of the government legislative initiatives. This, then, increases the need to try and balance the media debate. As we have discussed before, I have reserved time to be with you and to assist in the drafting of materials in response to media arising from the conference. I gather that Colin Goddard of Philip Morris Hong Kong has also stated that he could attend. I assume that we will need to set-up, in advance, a plan of action to include the details of conference monitoring, mechanisms of retrieving the information for the conference, and facilities for preparing briefs. I assume that we should be well prepared with Dr. Chatterjee and Dr. Springall monitoring the conference, and myself and perhaps Mr. Goddard working with you to prepare media materials. Again, please let me know if I can help in any way. | ttdf0194 |
| Jun-94 | Proposed Legislation | Amit Sarkar | Chris Proctor, BAT | Amit Sarkar writes to Chris Proctor saying that the copy of the legislation was not in public domain and shared key features available through parliamentary question. | ptdf0194 |
| Jun-94 | Questioning the data released by WHO | Amit Sarkar | Sharon Boyse | Whether WHO data on ad bans and consumption patters is irrefutable, particularly claims from New Zealand and Canada or statistical evidence to the contrary exist. Is it still possible to say that there is no conclusive evidence that there is a causative link between use of tobacco and incidence of diseases? | hzdf0194 |
| Jun-94 | Questioning the data released by WHO | Sharon Boyse, Manager Smoking Issues, BAT | Amit Sarkar | Any claims that advertising bans lead to decreased consumption of the product can certainly be challenged. See the BAT advertising brochure for references. It is more a question of challenging the interpretation of the data (in New Zealand and Canada) rather than refuting – the question is, did the decline in consumption have anything to do with advertising or it was due to tax increases, smuggling etc. | gzdf0194 |
| Jun-94 | TII newsletter | TII | Tobacco industry  Policy makers | Publishes letter send by 35 Members of Parliament addressed to the Prime Minister of India requesting a review of the proposed anti-tobacco bill. Publishes tobacco board resolution against the Bill and seeking its referral to an expert committee for a thorough examination, wider discussion with all stakeholders and keep the proposed law in abeyance till the completion of the process of consultation and recommendations of the committee. | ytdf0194 |
| Jun-94 | Indian Government plans tougher tobacco laws | BMJ | Public | Proposed tobacco control law ban smoking in public places, ban advertising, extend health warning to all tobacco products and ban sale within 100 metres of educational institutions. Industry launched a nationwide campaign to counter what it claims is “western oriented, anti-tobacco propaganda”. | hjpj0048 |
| Aug-94 | Lobbying against the proposed legislation to ban advertising of tobacco products in India | Norman Davis, Director BAT | John Holmes, Economic and Commercial Counsellor, British High Commission | Seeking support on serious BAT business interest in India. Comprehensive legislation proposed by Government (to be introduced in winter session of Parliament) would severely restrict marketing of all tobacco products including: • A total ban on all forms of advertising of all tobacco-related products • No advertising/stocking of tobacco products within 100 metres of academic institutions, hospitals and places of worship • More emphatic health warnings on packs • Introduction of penalties for those smoking in no-smoking areas Willing to discuss with the Government of India a code of advertising practice which would impose responsible voluntary restrictions on the tobacco industry. Seeking support in making representation on our behalf to the appropriate authorities to prevent this bill becoming legislation. I would also be grateful if the principles of a voluntary code could be conveyed to the Indian Government. | ypvp0201 |
| Apr-94 | Voluntary Code | TII | Tobacco industry | First draft of the TII voluntary code | yzpd0110 |
| Sep-94 | Invitation for a talk by Finance Minister Dr. Manmohan Singh | Arun Bhatnagar, The High Commission of India, Minister (Economics) | Norman Davis, Director,, BAT | Finance Minister to speak on the current state of Indian economic reforms on Friday 30^th^ September 1994. | qlyw0210 |
| Sep-94 | PARG | Tobacco Institute of India | Shabanji Opukah, BAT | The West Bengal and Himachal Pradesh State Assemblies have passed a resolution authorizing the Parliament to make a Law discouraging the use of tobacco and tobacco products in the States.  Leading business daily reports that the tobacco leaf is actually quite beneficial, reports a weekly. While some are harmful others can be used to make medicine for blood thinning and even a possible anti-AIDS drug.  ITC chairman part of business delegation which visited Vietnam with the Prime Minister P.V. Narasimha Rao.  ITC Chairman elected as Alternate President of the Associated Chambers of Commerce for 1994-95. | zqwd0194 |
| Sep-94 | Tobacco industry news | Tobacco industry BAT records | Tobacco industry | The legislation was opposed by farmers, exports, manufacturers and media. In addition Members of Parliament charged that the measures proposed will be counterproductive, ineffective and economically debilitating and the law was without adequate consultation and rushed through. The TII on ad ban said it severely discriminate against cigarettes, allowing cheaper tobacco products to grow unchecked as in absence of advertisements, choice will be price-determined and favour bidi and chewing tobacco etc. | rxcb0206 |
| Sep-94 | Public relation/lobbying by TII | Vikash Mehta of Bates India | David Aitken, BAT | The TII is doing a public relation job against the anti-smoking lobby. Informs David that the TII newsletters are sent to bureaucrats, industrialists, ministers, MPs and relevant decision makers. | tmvc0192 |
| Oct-94 | Lobbying against proposed legislation | John Holmes, Economic and Commercial Counsellor, British High Commission | Norman Davis, Director BAT | Thank you for your August letter. Was consulting colleagues in London on Government’s policy on the issue. Will be pleased to apprise the Indian authorities of the agreement in UK as a means of controlling advertising other than a complete ban.  Will also let know the authorities your willingness to discuss a voluntary code of practice. Actively lobbying with Indian Government to not introduce legislation to ban advertising is more difficult as the British Ministers take the view that it is for the states (of the European Union) to determine their own controls in the light of national circumstances. So it would not be consistent to make a formal representations to the authorities here. We will do what we can to help you within the approach I have outlined. | klyw0210 |
| Oct-94 | Lobbying against proposed legislation | Norman Davis, Director BAT | John Holmes, Economic and Commercial Counsellor, British High Commission | Thank you for your offer of help with communication with the Government of India. Sending summary of current UK Situation. Mr Shabanji Opukah will bring a full copy with him. UK agreement is very severe and we would not like to see it implemented in India. Since August, the advertising ban legislation seems to have been deferred for at least a year. Will be grateful if you could discuss the subject with Saurabh Misra of ITC. | hlyw0210 |
| Nov-94 | Tobacco workers threaten to launch stir | All India Bidi, cigar and Tobacco Workers Federation | Central Government | Appoint an expert committee including the workers representative to consider pros and cons of the proposed legislation | gxjp0209 |
| Nov-94 | Shackles on Mind or Freedom to Choose | TII publication | Tobacco industry Policy makers | Publishes appeals and opinions of people and organizations opposing the proposed law, mainly opposing ban on advertising: Adhip Kumar Sarkar, President the Indian Newspaper Society write to health secretary MS Dayal Prof. Paul P de Win, Director General World Federation of Advertisers write to AN Verma Principal Secretary to Prime Minister saying ad ban would be infringement of Article 19 of the UDHR. JC Chopra, Chairman of the Indian Society of Advertisers and Arun Nanda President Advertising Agencies Association of India writes to Bhaskar Ghose, secretary MoIB. Editorials by: The Hindu, “An ill-conceived Proposal on April 4 1994. The Asian Age “Buts and Ifs” on March 18, 1994. The Pioneer “People start smoking for vastly different reasons” on March 30, 1994. The Spectator “Puff of Prejudice” on February 12, 1994. The Times “Tobacco Advertising should not be banned” on January 12, 1993. Financial Express “It could cost the govt. dear” on September 9, 1994 and “Queering the Pitch” on April 24, 1994. Article in press: The Pioneer “Towards the last puff” on March 24 1994. Indian Express “Should there be a ban on cigarette advertising” on March 7, 1994. The Economic Times “Move to ban tobacco ads flayed” on October 12 1994 and “Smoke Sreams” on March 20, 1994. Business Line “Choking the Smoke” on April 7, 1994. The Observer “WHO is the Govt. to ban tobacco ads?” on October 26, 1994. BusinessWorld “The proposed ban on cigarette advertising is kicking up a rumpus” on March 9-22, 1994. Time “Around the World, people keep puffing away” April 18, 1994. Eminent People: Rani Jethmalani MJ Akbar Khuswant Singh in Hindustan Times “Dhumrapan Nishedh” on May 14, 1994. Vir Sanghvi in magazine Sunday “The Anti-smoking fascists” on June 5, 1994. | kzvv0194 |
| Nov-94 | PARG | A shyam | Shubanji Opukah | Govt. is pushing ahead in the direction of a complete ban on advertising of all tobacco products. The law which has been in making since 1986, when the exercise was begun by a committee of secretaries, is being given final touches by the Law Ministry. | tqwd0194 |
| Dec-94 | Lobbying against proposed legislation | John Holmes, Economic and Commercial Counsellor, British High Commission | Norman Davis, Director BAT | I wrote to the Ministry of Health a few weeks ago about their proposed tobacco legislation, suggesting the merits in British [ways] of a voluntary code and offering to discuss the issues or provide appropriate material. There has so far been no response. | pjyw0210 |
| Dec-94 | PARG | Tobacco Institute of India | Shabanji Opukah, BAT | Madhya Pradesh Government to ban chewing and smoking in government schools. | grwd0194 |
| Jan-95 | PR agency for BAT in India | Tom Watson, Managing Director, Halmark | Shabanji Opukah | Image inc recently advised the TII for PR on the ITGA meeting in Banglore. ITGA was impressed with the volume of press coverage obtained. Image Inc might be good alternative to a multi-national with all its baggage and they also have additional virtue of having some exposure to tobacco industry issues. | hjld0200 |
| Jan-95 | Issues raised in Parliament | TII | Tobacco industry Policymakers | Compilation of the key questions against the proposed legislation that ware raised in the parliament is shared with stakeholders. Amit Sarkar writing the preface for the booklet criticize the government stand that the decision were taken as per the discussions in the NCTOH. Calls for constitution of expert committee as suggested by the tobacco board of India | xsxy0191 |
| Feb-95 | Tobacco News, the TII newsletter against prposed legislation | TII | Tobacco industry policy makers | Publishes the joint petition made by trade unions to the Prime Minister, Health Minister and Labour Minister against the proposed legislation. Also publishes press coverage of this petition. Writes against WHO functioning saying it lacked analytical skill and political will. | tqdv0199 |
| Feb-95 | Internal tobacco industry communication | TII | Tobacco industry | Poll conscious Govt. defers tobacco ad ban bill. PM asked health and labour ministries to go slow until an alternative is not devised to compensate about 2 crore tobacco growers and others associated with the industry. Trade union and advertising industry voiced objection. ITC bought sponsorship rights of cricket world cup for 9million pounds to name the trophy as ‘wills trophy’, besides in-stadia ads of cigarettes would also be affected. Govt. agencies asked to assess revenue loss due to the legislation. (The Observer, Delhi Feb 24, 1995) | xqdf0194 |
| Mar-95 | PARG | Tobacco Institute of India | Shabanji Opukah, BAT | The Prime Minister is said to have asked the concerned Ministries to go slow on the legislation until an alternative is devised to compensate growers and others associated with it.  The Union Government proposes to introduce a Bill to ban smoking in public places and offices. The Central legislation once enacted can be adopted by the States through a resolution passed by the State legislatures.  In a media poll conducted by a leading national daily in New Delhi on whether smoking in public has an adverse impact on non-smokers an overwhelming 95% of the capital's denizens said it does and 86% were of the opinion that smoking should be banned in public places. | klgp0197 |
| May-95 | Proposed legislation and Parliamentary Committee Questionnaire | Amit Sarkar, Tobacco Institute of India | Shabanji Opukah, BAT | Help with response to questionnaire circulated by the Parliamentary Committee on Suppordinate Legislation. Request for visit to India by one team member perhaps Colin Goddard (from PMI). Shared presentation made by TII before the Health Ministry on 20 January 1995. | kkgp0197 |
| May-95 | Discussion on the Parliamentary Committee Questionnaire | Colin L Goddard | Christopher Proctor, BAT | Add to the discussion points the 18 questions circulated by the Parliamentary Committee on Subordinate Legislation in India. Next meeting proposed on 24 May 1995. We are trying to get it postponed. | zkgp0197 |
| May-95 | Voluntary Code – share US experience and RJR efforts against smoking by children | Amit Chaudhery | Thomas A McCoy | We would be immensely obliged if you could please send us copies of your campaign including all support information on voluntary code conduct / pamphlets / booklets / posters direct mailers etc. at your earliest convenience as we are pressed for time. | lmxj0094 |
| May-95 | Tobacco News TII newsletter | TII | Tobacco industry, policy makers | Publishes critique of WHO and an article on who benefits from WHO titled ‘what is wrong with WHO’. | tjlh0214 |
| May-95 | Help in responding to parliamentary committee on subordinate legislation | Amit Sarkar | Linda Rudge | TII invited by the COSL to discuss the cigarettes act 1975. colin  . Meeting is on 24th May 1995. | glgp0197 |
| May-95 | Help in responding to parliamentary committee on subordinate legislation | Rakesh Koul | Linda Rudge | List of points of discussion including: The introduction of rotational warnings  Symbols and figures (e.g. skull and cross bones) should be used as health warnings Printing of tar/nicotine contents on packs/ads and/or prohibition of sale to tobacco products to minors has helped to reduce consumption. Data/examples to show interchangeability in consumption between smoking and non-smoking tobacco proudcts. | flgp0197 |
| May-95 | Help in responding to parliamentary committee on subordinate legislation | Shabanjui Opukah | Amit Sarkar | Enclosed copy of voluntary agreement from Uzbekistan. Real author of the questions is the WHO Deal with the issues in line with our guidelines and policies. Use BATCo publications on these various subjcts. Brazil has the most recent experience where tobacco industry stymied the pictorial health warnings attempts, as against the freedom of commercial speech and unconstitutional in Brazil. We very much prefer that matters relating to the industry be dealt with by way of voluntary codes or agreements, instead of regulations from government. | skgp0197 |
| May-95 | India legislation questionnaire (COSL) | Shabanji Opukah | Chris Proctor David Bacon | It seems to me that in fact these questions may not have been meant for the industry. Sound very much like they are from the WHO to the regulatory authorities. The answers provided by the TII are largely irrelevant in most of the cases and also quite incriminating of the industry. There is also a very conscious effort on their part to appear to persecute the bidids, but this seems to work against the general grain of our industry argumentation on a number of issues. Do not progress in this manner. Don’t let any of these answers get out of their possession. | nkgp0197 |
| Jun-95 | RJR share documents on Voluntary Codes in US and efforts by them | Herbert E Osmon for Thomas A McCoy | Amit Chaudhery | The United States tobacco industry established a Cigarette Advertising Code in 1984 and a Code of Cigarette Sampling Practices in 1981 (amended in 1983). In 1990, these two codes were combined, with amendments, into the Cigarette Advertising and Promotion Code, which is the current and only voluntary code that has been adopted by the U.S. tobacco companies. Copies of the code are enclosed. As you read through the elements of the code, you will see that it clearly states our belief that cigarette smoking is an adult custom and that children should not smoke. It addresses specific behavior, activities, and conduct relating to ensuring that the advertising and sampling of tobacco products addresses adult smokers only. | zrnc0003 |
| Jun-95 | Questionnaire from the Parliamentary COSL | Amit Sarkar | Shabanji Opukah | Response from S Narain of GPI received. Waiting for your response. | smjb0207 |
| Jun-95 | Response to Parliamentary COSL | Shabanji Opukah | Amit Sarkar Chris Proctor Philippa Casingena Simon smith | In addition to the response to the COSL questions, BAT rebuts all the response presented by TII in their draft shared with BAT as mild and to be used by anti-tobacco advocates to their advantage. | pfmn0197 |
| Jun-95 | Response to Parliamentary COSL | Amit Sarkar | Shabanji Opukah | Hope you will expedite your response to the questions. Rohinton Mehta and I would like to come and visit to the tobacco manufacturers association, legal firms and tobacco documentation centre in addition to BAT departments. We would like this visit to happen before the next COSL meeting. | jljb0207 |
| Jun-95 | Amit and Rohinton visit to BAT | Shabanji Opukah | Amit Sarkar | You make all necessary arrangement for the visit. I am sending you the formal invitation. I would suggest that you should by way of your preparation, come ready to brief us on the main issues affecting the industry in India and what you think the future opportunities and challenges are. This would cover areas such as legislation, economic liberalisation, tobacco growing and the environment, smoking and health issues and taxation. You may think of other more or less important items for inclusion. Would you like me to arrange a media training course for one of the days? Please advise soonest to enable me fit it in the programme which I will send to you in advance . | skjb0207 |
| Jun-95 | COSL Meeting | Amit Sarkar | Shubanji Opukah | COSL meeting on 12^th^ July only Rohinton can come to London. I may not be able to accompany him. | mkjb0207 |
| July-95 | Constitution of Expert Committee | PP Singh | L Prasad | Ministry of Health constitutes an expert committee wide order no P.16016/1/95-PH on 26^th^ July 1995. | kjfk0208  gkfk0208 |
| Aug-95 | Tobacco News the TII newsletter | TII | Tobacco industry Policymakers | Editorial on right to advertise based on Supreme Court judgement in Civil Appeal No 6960 of 1994. Writes a critique of the Delhi Prohibition of Smoking and Non-Smokers Health Protection Bill, 1995 saying its provisions, methods of implementation and necessity is questionable. Sending the Bill to a committee is a positive step since the Bill appears to be guided by a lack of knowledge and biased interpretations of ETS. | hrdv0199 |
| Sep-95 | Dr. PP Singh visit to US | Collin Goddard of PMI writes for helping Dr PP Singh with meeting most appropriate persons in US who could help him. | Marc Firestone and Harris S Donald. | Dr Singh is the Industry’s representative on a Select Committee, set up by the government to look into tobacco and possible future legislation. We are working on a cooperative basis with the other members of the industry. | lxpn0010 |
| Oct-95 | Expert Committee on Economics of Tobacco | Linda Rudge, BAT | Dr PP Singh, ITC | Shares three volume of submission made by the UK Tobacco Manufacturers’ Association to the UK Governments’ scientific committee. | skvl0210 |
| Nov-95 | The International Tobacco Growers Association (ITGA) meeting in Bangalore | ITGA | Tobacco industry, tobacco farmers, policy makers | Chief Minister H. D. Deve Gowda openly took up cudgels for the industry and pleaded with the Centre not to impose any ban. | ksxy0191 |
| Dec-95 | Report of the Parliamentary Committee on Subordinate Legislation | Amal Datta, chairman | Parliament | Committee was overall in favour of a comprehensive legislation to prevent smoking in public places, total ban on all forms of advertisements of tobacco, statutory on all tobacco products and at all point of sale. | lmvl0210 |
| Jan-96 | Briefing on environmental tobacco smoke and commercial expression or ad ban | Amit Sarkar, Tobacco Institute of India | Chris Proctor, BAT | On 22nd and 25th January in Delhi for the Health Minister/MLAs of Delhi in connection with their proposed Prohibition of Smoking Bill. The Health Ministry's Expert Committee (General Health issues could also he covered in this interaction); selected Media persons, and, possibly, Union Ministers. Goa on 23rdJ24th January for a selected group of MP and Industry representatives.  PMI is likely to arrange for Dr Roger Walk_ a Scientist from Inbifo, Cologne (Germany) as a specialist resource for this. Seminal organised by the Advertising Club of Delhi at 6 00 PM on Monday 22nd January. It is planned to have 4 speakers- one each representing the Advertising Industry, the Media, the Legal Profession and a foreign expert on the ineffectiveness of ad bans. The specialist resource for this is likely to be Dr. John Luik of Canada who has been approached by PMI. | jskb0207 |
| Jan-96 | Briefing on environmental tobacco smoke and commercial expression or ad ban | Amit Sarkar, Tobacco Institute of India | Chris Proctor, BAT | Agenda with proposed meeting with Health Minster and MLAs of Delhi, Chairman of the Expert Committee and other officials of Health, Labour, Commerce, Finance, Industry and Public Environment shared. Video film to be made from interaction and presentation by John Luik, Chris Proctor, Eric Windholz and Roger Walk. | sqkb0207 |
| Jan-96 | TII voluntary code | TII | Tobacco industry | TII released a draft voluntary agreement regulating sales promotion activities and advertising of tobacco products. | smcp0099 |
| Jan-96 | Voluntary Code comments from Godfrey Phillips India ltd. | Ram A Poddar | Amit Sarkar | John Bevan Corporate Affairs Director of Rothmans, UK indicated that the Code is a good idea. Code is quite loose and needs to be made tighter especially: Add in Para 1: "1.3 Cigarettes are a legal product, and manufacturers have the right to inform their customers about their products" Definition 'Advertising' should include Point of Sale material and bill boards. Add to Para3: "No tobacco advertisement shall invole politics or religion". Rothmans desire to take membership of TII. | hmkb0207 |
| Jan-96 | PARG | A Syam | Shabanji Opukah | Delhi High Court issued show cause notice to Delhi govt. based on the PIL by Dr Rajesh Chawla. Health Minister remoulding the bill to be presented before the court. Dr Sharad Vaidya from Goa request President of India to not to inaugurate the Wills World Cup for Cricket as it was linked to tobacco use (WILLS). TII Director Amit Srakar tells media that international research has proved that sports events did not encourage any individual to smoke. | gykb0207 |
| Jan-96 | Voluntary codes proposed by TII | Tobacco News the TII Newsletter | Tobacco industry, policy makers | Voluntary code best way to regulated cigarette ads | jsxy0191 |
| Feb-96 | Letter to health minister on expert committee on the economics of tobacco | Rohinton R Mehta | Amit Sarkar | Letter approved by Mr Mishra from ITC sent by DR PP Singh to the Expert Committee. Similar letter should also be sent by TII to the chairman of the expert committee. Draft enclosed. | tpkb0207 |
| Feb-96 | Voluntary Code comments from BAT | Shabanji Opukah | Amit Sarkar | Agree with suggestions from John Bevan except for the definition. POS and Bill boards should be kept separate. Instead of “tobacco user”, which could be severly limiting to the industry and may in fact better serve the anti-tobacco lobbyists, just refer to “people above the voting age”. In 7.3. why not simply state; " Tobacco companies shall ensure that any co-sponsors using their brands will adhere to this code in so far as it covers tobacco sponsorship ." In 8.2 define what we mean by Members. I,e members of TII or all engaged in tobacco trade. In 9.2 add the words, "or by entrusting the task to a third party organization agreed to by members of the Institute ." Delete last para of 10.1. | slkb0207 |
| Feb-96 | PARG | A. Syam | Shabanji Opukah | A voluntary self-regulation code for the marketing of tobacco proudcts, as an alternative to the proposed central ban on all commercial advertising, has been drawn up by TII. Tobacco advertising shall not claim ‘health properties’ for tobacco, will not be aimed at minors, no person under age of 25 years to be depicted in commercials, no politics or religion, primarily intended to effect brand switching, maintaining brand loyalty or induce inter-brand category shift among tobacco user who are above voting age. Dr. Sharad Vaidya of NOTE from Goa write and appeal to Lata Mangeskar, Pandit Jasraj and Zakir Hussain to not take part in the WILLS Cricket World Cup inaugural function. Chairman of the Parliamentray Committee Amal Datta told media that although cigarette ads are banned on Doordarshan, beaming Wills ads to viewers throughout the sub-continent during the Wills World Cup would violate the policy. | qkxd0204 |
| Mar-96 | Voluntary Code definition of POS | Amit Sarkar, TII | Shabanji Opukah, BAT | Directors want to know: The appropriate definition and wording under which POS/Billboards should be shown in the Code Whether exclusion of the above two items from the heading "Advertising" (as currently featured in the draft Code) or, complete exclusion from the Code would imply that they would not, subject to certain stipulations like featuring the statutory warning, etc If not, then how would the Code cover this aspect? Our activity is not meant to expand the total tobacco market, but only to create brand switch. Also awaiting comments from Colin Goddard | hhmf0195 |
| Mar-96 | Draft letter to Chairman Expert Committee on economics of tobacco | Amit Sarkar | K.N. Kabra | Except three members others are not experts on economics. Constitution of the committee is basically flawed with seven health experts and three experts on economics Srinath Reddy and PC Gupta are extremist anti-tobacco | qjjp0197 |
| Mar-96 | Draft letter to health minister on expert committee on the economics of tobacco | Amit Sarkar | A R Antulay, Union Minister for Health and Family Welfare | In 12 member committee seven are medical/health specialist and economists only two making the composition of the committee unbalanced. | pjjp0197 |
| Mar-96 | Comments for submission to expert committee on economics of tobacco | Amit Sarkar | Directors TII and its constituent companies Shubanji Opukah PP Singh R Mehta | Views of Eric Windholz from PMI shared and seeking inputs in one week for submission to the committee. | rpkb0207 |
| Mar-96 | Voluntary Code, definition of POS | Shubanji Opukah | Amit Sarkar | The word POS stands alone and in relation to the actual location where the industry wishes to see advertisements placed. This would actually define these locations eg kiosks, bars, hawker tables, supermarkets, shops etc etc . I would suggest that we do the same with the Indian code. As for Billboards, my suggestion is that we should perhaps leave them out since including them might invite restrictions on what in fact we traditionally regard as not qualifying for inclusion under the definition of advertising. They may however be subject to HWCs as is the case in many markets already. | rlkb0207 |
| Mar-96 | Election in India | Shabanji Opukah | Amit Sarkar | I believe the whole country is quite busy with the electioneering and thankfully there seems to be some reprieve on the industry front. | frkb0207 |
| Apr-96 | Supreme Court of Canada judgement and legal opinion of Indian legal luminaries on ad ban law for Law Ministry | Rohinton R Mehta, ITC Ltd. | KS. Vaidyanathan, ITC Ltd. | Pursuant to Review Meeting of April 5, 1996 please find documents which need to be passed over to the Ministry of Law. Summary and complete Judgment of the Supreme Court of Canada Synopsis and complete opinion of Indian legal luminaries on the constitutionality of a tobacco advertisement ban legislation. | yzyh0206 |
| Apr-96 | Legal opinions on ad ban by legal luminaries | TII | Tobacco industry Policymakers | Opinion of sis legal luminaries on ad ban: 1) Hon. Justice P.N. Bhagwati - Former Chief Justice, Supreme Court of India. 2) Hon. Justice Y.V. Chandrachud - Former Chief Justice, Supreme Court of India. 3) Hon. Justice E.S. Venkataramiah - Former Chief Justice, Supreme Court of India. 4) Hon. Justice Bakhtavar Lentin (Retd .) - Bombay High Court. 5) Phiroze R. Vakil - Senior Advocate, Bombay High Court. 6) Prof. T. Devidas - Principal, M.S. Ramaiah Law College, Bangalore. | grfm0208 |
| Apr-96 | PARG | A. Syam, Executive Vice President Public Relations, TII | Shabanji Opukah | The Punjab Vidhan Sabha has passed a resolution recommending to Parliament the enactment of a legislation to ban the use of tobacco in public places in the State and a ban on all forms of advertising relating to tobacco products. | ylkb0207 |
| May-96 | Shabanji India vist including WNTD and proposed legislation | Shabanji Opukah | Tony Johnston Andrew Napier Simon Smith | Parliamentary Bill to ban all tobacco advertising – Colin Goddard PMI Hong Kong and head of ITC tobacco division attended – it was agreed that there still remains a real threat in spite of the recent political changes in India. TII will put in motion the lobbying strategy that BAT helped them put together. Appointed PR agency and an economists to be utilized in communications and lobbying on the bill. Delhi smoking prohibition Bill – withdraw the bill or have amended in line with the voluntary code proposals. I suggested to use the courtesy of choice campaign programme which CORA is working to introduce in some of our markets. India Voluntary Code of Advertising – This is one of the initiatives in place addressing the threats to marketing freedom in India. We discussed and agreed on its contents and the communication strategy. WNTD – We discussed the initiatives proposed for managing this WHO orchestrated event. Highlighted the need for care in how PR consultancy, Speakers, TIIs involvement in such seminar. | hmhk0208 |
| May-96 | TII efforts against government tobacco control projects | Sankar De | David Bacon | Anti-tobacco legislation: Health ministry introduced draconian law banning advertising. WHO maintained sustained pressure on this aspect. As a result COSL headed by Amal Dutta constituted to consider the proposed law and make recommendations. The Cigarette industry realizing the import or this Committee impressed on Government that for a truly objective and fair assessment of the alleged effects of tobacco use, the economics of tobacco use should he-viewed in balance. The support from Ministries of Finance- Commerce, Agriculture, and Labour were garnered and an Expert Committee was constituted to evaluate the effects of tobacco use versus the economic benefits, When the constitution of this Committee revealed that they were comprising of representatives predominantly from the health fraternity and a few economists the Cigarette industry drove home the point that the Expert Committee did not equitably and adequately represent essential segments of the tobacco economics i.e. growers, labour. traders, and industry. Consequently, the scope and size of the Expert Committee was expanded with some representation from each of these segments.  Specific strategies were also executed by proposing some realistic and pre-emptive measures i.e. adoption of voluntary code on advertising and sponsorship. Giving publicity to the judgment of Canadian and Indian Supreme Court on freedom of commercial speech. Mobilising support form legislators, tobacco farmers and trade unions on dire consequences of proposed govt. measures. | mlkb0207 |
| Jun-96 | PARG | A. Syam, Executive Vice President Public Relations, TII | Shubanji Opukah | Tobacco farmers from Karnataka and Andhra Pradesh urge Prime Minister HD Deve Gowda to create a stabilization fund to be managed by the Tobacco Board to protect farmers from violent fluctuations in prices from year to year. | nkkb0207 |
| Jul-96 | Representation from ITGA to Prime Minister of India | H Gralow, President ITGA | HD Deve Gowda, Prime Minister of India | Writes about meeting in Bangalore in November 1995. Call for compromise with tobacco policy balancing the immediate socio-economic benefits for rural areas. Balance the interest of the tobacco sector. Proud of TII and hope balanced tobacco policies can prevail in India. | rmmv0207 |
| Jul-96 | IAA workshop on should cigarette ads be banned | Amit Sarkar | TII Directors | The Advertising Standards Council of India also took the latter stance and stated that they are willing to help in devising and progressing the Voluntary Code. We will be pursuing this with ASCI, reference the attached letter. The two agency speakers took up the cause of freedom of commercial expression. Mr. Bose, the moderator and convenor of the Workshop, also screened a portion of a video film sent by Carla Michelotti of IAA which was very supportive. In conclusion, whilst the medical antis were adamant in their recommendation for an ad ban, the prevailing opinion seemed to support a Voluntary Code/reasonable restrictions approach. The antis expressed the allegation, in private, that the Workshop appeared to have been stage-managed by the Industry. | fhgb0209 |
| Aug-96 | Tobacco advertising report | Joanne Swatten, Corporate Affairs Dept. BAT | Amit Sarkar | Shared the ‘Report on tobacco advertising-give children a chance’ comprising of the tobacco industry views on tobacco advertising and how to counter such laws. | hggb0209 |
| Aug-96 | Increasing investment in India Takeover bid | BAT Standing Committee | Board BAT | Company’s primary objective is to re-establish management control over ITC LTd. And that it is necessary to increase the shareholding from current 31.4%. | hfyb0209 |
| Sep-96 | Constitution of Expert Committee on Economics of tobacco | Health Ministry | All stakeholders | To undertake a comparative study on the economics of tobacco use inter-alia examining the tax revenue and foreign exchange earnings, employment and consumer expenditure on the one hand and the cost of tertiary level medial care facilities for treatment of tobacco related diseases, losses due to fire hazard, ecological damage due to deforestation, and disposal of tobacco related waste on the other hand with a view to making an economic study of the impact of tobacco consumption. Committee headed by K.N.Kabra Other members included PC Gupta, KS Reddy, representative of tobacco board, director CTRI, Indian tobacco association, Indian national trade union, farmers and tobacco grower’s association etc. and Dr Kishore Chaudhary of ICMR as member secretary. | lxvl0210 |
| Oct-96 | Meeting on India CORA/PR project | Shabanji Opukah, BAT | Tim Lord, BAT | Agreed on need to build relations between ITC and BAT and BAT to transfer best CORA practice to ITC. SO already working on this and Ranjit Jacob is to visit in December for induction. Agreed on need for close BAT liaison with TII. Tim Lord to ask Malcolm Fry to attend meetings. SO arranging for TII Director, Amit Sarkar, to visit for work on the Expert Committee responses with CORA in London. | gtfb0209 |
| Oct-96 | Seeking published data from BAT | Amit Sarkar | Shabanji Opukah | Seek your advice in identifying and obtaining published data on an international level, based on your own knowledge as well as recommendations from Dr PP Singh/Rohinton to be submitted to Expert Committee. | kxvl0210 |
| Oct-96 | Appreciation of BAT and PMI | Amit Sarkar | Colin Goddard Shabanji Opukah | The Board expresses its appreciation to PMI and BAT for making available the services of experts on the subject of advertising ETS and Legal issues concerning the Industry, for interactions in India. | psfb0209 |
| Oct-96 | Expert Committee framework | Amit Sarkar | Directors TII | Approaching BAT for comments on the proposed framework and perhaps Mr Narain could do the same in respect of PMI. TII addition in framework: Alleged addictive nature of tobacco the costs are voluntary and not personal On the benefit sides, savings on pension due to premature death Even if tobacco was not there, people will ultimately get sick and will anyway incur the same expenses on the treatment sooner or later. | qhvl0210 |
| Oct-96 | Expert Committee submissions | Amit Sarkar | Rohinton Mehta | Submit TIIs publication on tobacco taxation and parliament questions and answers, 22^nd^ report of the COSL establishing the economic importance of tobacco and survey report on Health Needs of the 21^st^ Century” a study of minsters of health in 67 developing countries by the Institute for International Health and Development, USA. | phvl0210  tqdy0044 |
| Oct-96 | Letter by Shiv Sena MP to Expert Committee | Satish Pradhan, MP | Salim Sherwani, Minister of Health Expert Committee on economic of tobacco use | In capacity of sports lover and as president of Maharashtra Amateur Athletic Association and Maharashtra State Table Tennis Association.  Tobacco industry is one of the largest and most consistent promoters of sports and sport development in India. Some conclusions of the Committee will adversely affect tobacco advertising promotion and sponsorship in India. this would be serious loss. Since sports will be seriously impacted we would request that in all fairness a nominee of our organization be included in the above committee. | mzcx0206 |
| Oct-96 | Letter by MP to Expert Committee | Suresh Kalmadi, MP | Salim Sherwani, Minister of Health Expert Committee on economic of tobacco use | In capacity of sports lover and as president of Amateur Athletic Federation of India (AAFI).  Tobacco industry is one of the largest and most consistent promoters of sports and sport development in India. Some conclusions of the Committee will adversely affect tobacco advertising promotion and sponsorship in India. this would be serious loss. Since AAFI will be seriously impacted we would request that in all fairness a nominee of our organization be included in the above committee. | nzcx0206 |
| Oct-96 | Evaluation of model proposed to be used by Expert Committee | Amit Sarkar | Farrell Delman President Tobacco Merchant’s Association of the US | We wonder whether this could be done by Wharton Econometric Forecasting Associates (WEFA) which is an acknowledged institution whom you used for your annual economic analysis or, any other suitable person/institute. | lzcx0206 |
| Oct-96 | Components of economics of tobacco | Kishore Chaudhry Member Secretary | PP Singh, ITC Ltd. | Find enclosed the identified components regarding economics of tobacco. As decided in the meeting please send your suggestions and data, if any, at an early date. | lzcx0206 |
| Jan-97 | Details of discussions with BAT in UK with David Bishop Christopher Proctor Philippa Casingena Lenny Abelman Andrew Napier Tony Johnston Shabanji Opukah | Amit Sarkar, TII Rohinton Mehta Dr PP Singh | Chairman TII | Point needs to be made to the Expert Committee that personal benefits to smokers are not "only intangible” as suggested in their "Framework" but can be measured through elasticity studies which determine the consumer surplus" accruing to therm. Further critiques of the above 'Framework" and an evaluation of the economic impact studies done by TH will be undertaken by Ernst & Young and sent to us. Their economist Mr. Tom McClean may also be made available for further interaction with us at a cost. Whether there should be legal action regarding ad ban. (Examine the ad ban cases lost by alcohol industry, to see whether similar arguments used); (b) What action to be taken to explain ETS/Self- Regulation concept to other States likely to follow suit (BJP States ; UP/WB/Goa/Kerala/AP which have passed resolutions endorsing the propose Central Legislation). legal action proposed, then what is the best timing : immediately, or after extension to other States (select); or, after Central Legislation | qrfb0209  rplx0195 |
| Jan-97 | Thank you for interaction | Amit Sarkar | Lenny Abelman, BAT | Thank you for sparing time to interact on matters relating to Expert Committee, PILs etc during our recent visit which will be of tremendous importance in enabling us to tackle these issues effectively. | hzgd0204 |
| Jan-99 | PMI Report on recent international developments Advertising and sponsorship in India | PMI | PMI | ASCI withdrew its proposed tobacco advertising code as tobacco companies were unable or unwilling to comply with it. TII told ASCI it had developed its own tobacco advertising code, which it intend to follow. | jgwb0008 |
| Mar-99 | India policy and legislation | Tobacco Journal International | Tobacco Industry | Economic liberalization under PV Narasimha Rao and Manmohan Singh generally beneficial to local manufacturers. However, benefits have been tempered by a number of tax increases and exacerbated by the proposed legislation which would ban tobacco advertising and sponsorship. Arrival of foreign brands on the domestic market has further increased competition. | yzkl0212 |
| Nov-99 | Expert Committee Report | Amit Sarkar | R.A Poddar | Global tobacco control law: Towards a WHO FCTC meeting in New Delhi from 7-9 January 2000. List of participants for information. | jgjp0206 |
| * Tobacco industry documents can be accessed at https://www.industrydocuments.ucsf.edu/docs/#id=[reference number] | | | | | |

| **Table S3: Key players in the tobacco industry and government and their roles (1990–1999)** | |
| --- | --- |
| **Name** | **Position/purpose** |
| **Key organizations/departments in the tobacco industry** | |
| British American Tobacco (BAT) | A 1902 British multinational tobacco company headquartered in London with the highest foreign stake in India’s cigarette market (British American Tobacco Limited, 2020). |
| Corporate Regulatory Affairs Group (CORA) | A department in BAT “to protect and strengthen the business performance of BAT” |
| Public Affairs Resource Group (PARG) | Formed in 1992 by BAT to manage issues of public concern related to tobacco and health and coordinate public relations efforts with regional coordinators. |
| Tobacco Institute of India (TII) | Formed in November 1992 on the initiative of the three leading Indian cigarette companies i.e. Godfrey Phillips India Limited, ITC Limited and VST Industries Limited to act in the interests of the Indian cigarette and cigarette-tobacco industries. It replaced the by then dismantled, Cigarette Manufactures’ Association (Unknown, 1994a). |
| ITC Limited | A 1910 Indian multinational tobacco company with highest market share of cigarette sales in the country. As of 2019, nearly 30% of company shares are owned by the British American Tobacco (Saha, 2019). |
| Godfrey Phillips India Limited (GPI) | An Indian tobacco company with the second highest market share of cigarettes in India. It was nearly 100% owned by Phillip Morris during the 1970s but as of 2019 the Phillip Morris Asia International owns 25.1% shares of the company (Business Standard, 2019). |
| **Tobacco industry personnel** | |
| Norman Davis | Director, BAT |
| David Bacon | Head, Public Affairs/Corporate Communications/Corporate Affairs, BAT |
| Shabanji Opukah | Head, International Development Issues; Regional Coordinator, East African PARG; Manager, CORA, Africa, Middle East, South and Central Asia, BAT |
| Fran Morrison | External Communications Manager-CORA, BAT |
| Chris Proctor | Chief Scientific Officer, BAT |
| Sharon Boyse | Manager Smoking Issues, BAT |
| Digby Anderson | Director Social Affairs Unit, BAT |
| Linda Rudge | Smoking Issues Department, BAT |
| Collin L Goddard | Director, Environmental Policy, Worldwide Regulatory Affairs, Phillip Morris International |
| Thomas A McCoy | Senior Vice President – External Relations RJ Reynolds Tobacco Company (Bought by BAT in 2017) |
| F. M. Lenny Abelman | Regional General Counsel, BAT |
| Simon Roper | Legal and Secretarial Department, BAT |
| Ferrell Delman | President, Tobacco Merchants’ Association of the US Inc. |
| Eric Windholz | Director, Worldwide Regulatory Affairs, Asia/Australia/Japan Phillip Morris International |
| John Bevan | Corporate Affairs Director of Rothmans, UK |
| Amit C. Sarkar | Director, TII |
| Amit Chaudhery | General Manager, Communications, TII |
| Rakesh Koul | TII Employee |
| A. Syam | Executive Vice President Public Relations, TII |
| Rohinton R Mehta | Corporate Legal, ITC Ltd. |
| K. S. Vaidyanathan | General Manager Corporate Affairs, ITC Ltd. |
| Ram A Poddar | Chief Executive, GPI |
| S Narain | Advisor Corporate Affairs GPI |
| K L Chugh | Chairman ITC Ltd. |
| Dr PP Singh | Manager, Integrated Research Laboratory, ITC |
| Sankar De | Manager Public Affairs, VST Industries Limited |
| **Government and public health personnel** | |
| P.V. Narshima Rao | Prime Minister of India (21 June 1991 – 16 May 1996) |
| H. D. Deve Gowda | Prime Minister of India (01 June 1996 – 21 April 1997) |
| Man Mohan Singh | Union Minister of Finance |
| John Holmes | Economic and Commercial Counsellor, British High Commission |
| M. L. Fotedar | Union Minister of Health and Family Welfare |
| Arun Bhatnagar | Minister (Economic), High Commission of India, London |
| R.L. Mishra | Secretary, Ministry of Health and Family Welfare |
| Tushar Kanti Das | Joint Secretary, Ministry of Health and Family Welfare |
| K.P. Unnikrishnan | Deputy Secretary (PH), Ministry of Health and Family Welfare |
| **Retired judges, senior advocates and academicians** | |
| P.N. Bhagwati | Former Chief Justice of India |
| Y.V. Chandrachud | Former Chief Justice of India |
| E.S. Venkararamiah | Former Chief Justice of India |
| Bakhtavar Lentin | Retired Judge Bombay High Court |
| Phiroze R. Vakil | Senior Advocate, Bombay High Court |
| T. Devidas | Principal, M.S. Ramaiah Law College, Bangalore |

| **Table S4: Organizations and experts who submitted their comments and suggestions to COSL**(1995) | | |
| --- | --- | --- |
| **Organizations** | **Affiliation** | **Area of expertise** |
| 1. Ministry of Health and Family Welfare (Department of Health), New Delhi | Government | Health |
| 2. Indian Council of Medical Research (ICMR), New Delhi | Government | Health |
| 3. World Health Organization | Inter-governmental | Health |
| 4. Indian Cancer Society, New Delhi | Non-governmental | Health |
| 5. Dr. S.G. Vaidya, Goa Cancer Society, Goa | Non-governmental | Health |
| 6. Sitaram Bhartia Institute of Science and Research, New Delhi | Non-governmental | Health |
| 7. Dr. G.K. Rath, All India Institute of Medical Sciences | Government | Health |
| 8. Dr. R.P. Sapru, Post Graduate Institute of Education and Research, Chandigarh | Government | Health |
| 90. Dr. S. Krishnamurthi, Cancer Institute, Madras | Government | Health |
| 10. Dr. V. Raman Kutty, Health Action by people, Trivandrum | Nongovernmental | Health |
| 11. Ministry of Human Resource Development (Department of Education), New Delhi | Government | Education |
| 12. Maruti Sewa Samiti, Udaipur | Non-governmental | Social service |
| 13. Upbhokta Seva Sangh, Bihar | Non-governmental | Consumer rights |
| 14. Indian Institute of Consumer Studies, Bangalore | Non-governmental | Consumer rights |
| 15. Consumer Education and Research Centre | Non-governmental | Consumer rights |
| 16. The Indian National Trade Union Congress New Delhi | Non-governmental | Trade |
| 17. The Tobacco Institute of India, New Delhi | Non-governmental | Tobacco industry |
| 19. The Indian Tobacco Institute, New Delhi | Non-governmental | Tobacco industry |
| 19. The Indian Tobacco Association, Guntur | Non-governmental | Tobacco industry |
| 20. The Andhra Farmers Forum, Rajamundry | Non-governmental | Tobacco farmers |
| 21. East Godavari District Tobacco Growers and Farmers Association, Andhra Pradesh. | Non-governmental | Tobacco growers |
| 22. The Kalinga Beedi Workers Forum | Non-governmental | Bidi workers |
| 23. All India Beedi, Cigar and Tobacco Workers Federation, Maharashtra | Non-governmental | Tobacco workers |

| **Table S5: Comparison Cigarettes Act 1975 and the proposed law of 1994** | | |
| --- | --- | --- |
| **Key provisions** | **Cigarettes Act 1975** | **Proposed Law 1994** |
| Ban on smoking in public places | No provision | Prohibition on smoking in public places. |
| Ban on sale to minors | No provision | Prohibition on sale of tobacco and tobacco products to minors and near education and health institutions. |
| Ban on advertising and promotion of tobacco products | No provision | Ban direct and indirect advertising and sales promotion of tobacco and tobacco products. |
| Health warning labels | 3 mm text warning i.e. “Cigarette smoking is injurious to health” on cigarette packs and advertisements | Any of the following statutory warnings on all packages of tobacco and tobacco products in appropriate forms, sizes and languages to make these prominent and effective.   - 'Smoking can lead to Oral Cancer ' - 'Smoking can cause heart problems' - 'Smoking may reduce your life span' - ‘Smoking can aggravate respiratory problems’ |

| **Table S6: Key Recommendations of the NCTOH July 1991 and the Tobacco Institute of India’s Response** | | |
| --- | --- | --- |
| Issues | NCTOH Recommendation (Unknown, 1992) | Tobacco Institute of India response |
| National Tobacco Control Commission | Establish a National Tobacco Control Commission (NTCC) to plan, coordinate and monitor tobacco control activities. |  |
| Ban on smoking and consumption of tobacco products in public places | a. Prohibition on smoking in public places.  b. Ban consumption of tobacco and tobacco products in public offices, transport facilities, restaurants, sports areas, museums and libraries.  c. Ban sale of tobacco and tobacco products to minors and near educational and health institutions and on at least one day a week at every other place. | The growing, manufacture and sale of tobacco products is an activity permitted by law and supported by Ministry of Agriculture and Ministry of Commerce. Restriction on use of legal product violates individual liberties. |
| Ban on advertising and promotions | Ban direct and indirect advertising and sales promotion of tobacco and tobacco products. | Completely erroneous belief that advertising expands products use.  Tobacco is a mature product that has been sold for three centuries. Advertising merely helps switch brand and ensure competition between brands.  Many studies have proved this and the Quebec superior Court has said that ban on tobacco advertising is not health legislation.  Alcohol use in India has increased between 1982-89 during which an advertising ban has been in place. |
| Health warning labels | Statutory warnings on all packages of tobacco and tobacco products and prescription of appropriate forms, sizes and languages to make these prominent and effective. | Warning labels only on cigarettes, pan masala and chewing tobacco, this is discriminatory and should apply to bidis and snuff as well.  Tobacco users in the country already aware of the health concerns and exercised their adult choice in favour of tobacco use.  Statutory health warning on packs have been considered adequate in US. Sweden rejected trebling the size of the warnings. Too many labels dilute the message and exaggerated warnings tend to trivialize the message. |
| Tar and nicotine levels | Printing of nicotine and tar content on all packages of tobacco products, their progressive reduction, and establishment of a national laboratory for licensing, testing and monitoring. | Such legislation would be premature in a country, where 80% of tobacco use in the form of biris, zarda and snuff, which would make evaluation and enforcement impossible.  Union Minister of State for Health had informed Lok Sabha that tar and nicotine levels are much lower in cigarettes compared to biris.  The Ministry of Health must guard against introducing discriminatory laws which focus on cigarettes alone, which are the most modern form, and constitute only 20% of tobacco consumption in India. |
| Licensing of tobacco products | Compulsory licensing of all tobacco products. | This runs counter to the Government's declared programme of liberalization, and the transition to a market-driven economy, free from Government control. Indeed, the licensing of cigarette capacities discriminates against the most modern form of tobacco use, and has no precedent in other free economies. |
| Environment protection | Compulsory afforestation (planting trees in barren lands to create forest) by tobacco producers and tobacco industry and prohibit use of wood for making paper for rolling cigarettes and packing tobacco products. | None |
| NGOs for monitoring and enforcement. | Penalties for violations of law and enable NGOs and legal activists to monitor and enforce law and launch public interest litigation. | None |
| Tobacco in toothpaste | Ban use of tobacco in toothpaste and toothpowder. | None |
| No increase in tobacco cultivation/production | Ban on further expansion of tobacco cultivation, manufactured tobacco products and on introduction of new tobacco products. | This suggestion ignores the economic impact of tobacco on the national economy and its enormous potential to contribute to exports, revenue, dryland farming and a wide range of ancillary industries dependent on it.  Tobacco use 0.3% arable land but yields1.0% agri-value, 4.0% of Central Government revenue and 5% of all agri-exports, provides livelihood to 1 million small farmers, 5 million farm labour 1.2 million self-employed small trader in retail, and wholesale trade besides ancillary businesses. Andhra Pradesh Chief Minister, Union Agriculture Minister and Food and Agriculture Organization has emphasized the economic importance of tobacco in the farming community. |
| Restriction on credit to tobacco growing | Ban on subsidies and restrictions on credit, for tobacco cultivation and processing. |  |
| Incentive to switch away from tobacco growing | Monetary help to farmers for changing over from tobacco cultivation to alternative crops. |  |
| Curtail investment of government funds in tobacco | Withdrawal of government holdings in tobacco industry. |  |
| Rehabilitation of tobacco workers | Economic and agro-industrial restructuring measures should be taken including rehabilitation of workers employed in tobacco industry. |  |
| Progressive increase in taxation on all tobacco products | Progressive increase in taxes on all tobacco products to the highest level and use of tax revenues for tobacco control activities by crediting these to a fund to be utilized by the National Tobacco Control Commission (NTCC) for health education, research, rehabilitation and other tobacco control activities. | This recommendation is completely at variance with the New Economic Policies of the Government of India, and the final recommendations of the Tax Reforms Committee headed by Dr Raja J. Chelliah, which clearly stipulate the need for long-term stability in tax rates and structure, recognizing that this will expand the tax base, and increase Government revenue.  Cigarettes contribute 90% of tobacco revenue, though only 20% of tobacco consumption. The balance 80%, comprising biris/zarda/snuff, contribute only 10% of revenue from the sector. The only efficient way to bring this 80% into the tax net is by keeping excise rates/structure on cigarettes constant and progressively expanding cigarettes’ share of tobacco consumption. |
| Social costs of tobacco use | Maximum emphasis should be givento health education to combat the menace of tobacco.  NGOs, especially working for children and women to play important role in health education and enforcement of tobacco control laws.  Further research and studies to strengthen tobacco control. ICMR to collaborate with NTCC in this work. | India is the only country in the world where 80% of tobacco consumption is virtually outside the tax net, and not contributing to Government in proportion to its sale. This can be corrected only by bringing them into the tax net, by converting to the cigarette sector.  It is simply not possible to have a credible analysis of the social cost in a country where primary health issues are inadequate e.g.  (a) 30% of the population is under-nourished 'being below the poverty line.  (b) 30% of the rural population is exposed to water-borne diseases for lack of potable drinking water  (c) over 90% of those living in villages lack basic sanitation  (d) mortality rate of children below the age of 5 years is a tragic 14% (e) AIDS is assuming epidemic proportions .  It is due to these factors that India has been ranked 121st, out of 160 countries, in the 1992 Report published by the UNDP on Human Development, based on the Health Profiles of Nations. |
| National action plan | Prepare a national tobacco control action plan based on these recommendations for implementation in phases at national, state and district level. | None |
| White paper on tobacco control policy | A white paper on government policy on tobacco should be prepared and laid before the Parliament. | None |

| **Table S7: Letters, Editorials and Opinions against the proposed national law (Sarkar, 1994)** | | |
| --- | --- | --- |
| **Date** | **Name and designation of the person who wrote the letter** | **Letters addressed to /editorials/opinions** |
| 22 August 1994 | Adhip Kumar Sarkar, President the Indian Newspaper Society | MS Dayal, Secretary, Ministry of Health and Family Welfare, Government of India |
| 31 March 1994 | Prof. Paul P de Win, Director General World Federation of Advertisers | AN Verma Principal Secretary to Prime Minister of India |
| 9 March 1994 | JC Chopra, Chairman of the Indian Society of Advertisers | Bhaskar Ghose, Secretary, Ministry of Information and Broadcasting, Government of India |
| 20 June 1994 | Arun Nanda President Advertising Agencies Association of India | Bhaskar Ghose, Secretary, Ministry of Information and Broadcasting, Government of India |
| 4 April 1994 | The Hindu | “An ill-conceived Proposal on |
| 18 March 1994 | The Asian Age | “Buts and Ifs” |
| 30 March 1994 | The Pioneer on | “People start smoking for vastly different reasons” |
| 12 February 1994 | The Spectator | “Puff of Prejudice” |
| 12 January 1993 | The Times | “Tobacco Advertising should not be banned” |
| 9 September 1994 | Financial Express | “It could cost the govt. dear” |
| 24 April 1994 | Financial Express | “Queering the Pitch” |
| 24 March 1994 | The Pioneer | “Towards the last puff” |
| 7 March 1994 | Indian Express | “Should there be a ban on cigarette advertising” |
| 12 October 1994 | The Economic Times | “Move to ban tobacco ads flayed” |
| 20 March 1994 | The Economic Times | “Smoke Sreams” |
| 7 April 1994 | Business Line | “Choking the Smoke” |
| 26 October 1994 | The Observer | “WHO is the Govt. to ban tobacco ads?” |
| 9-22, March 1994 | Business World | “The proposed ban on cigarette advertising is kicking up a rumpus” |
| 18April 1994 | Time | “Around the World, people keep puffing away” |
|  | Rani Jethmalani, Advocate, Supreme Court of India, well known women’s issues lawyer (opinion given to TII) | It does not make sense to have a complete advertising ban. People will still go and buy cigarettes. |
|  | MJ Akbar Editor of Asian Age and Telegraph newspapers and a former Member of Parliament (opinion given to TII) | How can you ban advertising of a product whose sale is permitted by the Government |
| 14 May 1994 | Khuswant Singh in Hindustan Times (Newspaper) | “Dhumrapan Nishedh” |
| 5 June 1994 | Vir Sanghvi in Sunday (Magazine) | “The Anti-smoking fascists” |

| **Table S8: Members of the Expert Committee on Economics of Tobacco appointed by the Ministry of Health** | |
| --- | --- |
| **Appointed on 26 July 1995 (Sarkar, 1995)** | **Expertise** |
| Prof. K.N. Kabra - Chairman, Indian Institute of Public Administration, Indraprastha estate, New Delhi | Economics |
| Dr. A.K. Kundu, Deputy Director-General (P) | Health |
| Director, V.P. Chest Institute, University of Delhi | Health |
| Dr Prakash C Gupta, Senior Research Scientist, Tata Memorial Institute for Fundamental Research, Bombay | Health |
| Dr K.S. Reddy, Professor of Cardiology, All India Institute of medical Sciences, New Delhi | Health |
| Dr N Anantha, Director, Kidwai Memorial Institute of Oncology, Bangalore | Health |
| Dr Sukhdev Thorat, Additional Professor of Economics, School of Social Sciences, Jawaharlal Nehru University | Economics |
| Prof. M.N. Reddy, National Institute of Financial Policy and Planning, New Delhi | Economics |
| Representative of Tobacco Board, Ministry of Commerce | Tobacco industry |
| Director, Central Tobacco Research Institute, Rajamundry, Andhra Pradesh | Tobacco industry |
| Representative of Indian Tobacco Association Virginia house, 37, Chauringhee, Calcutta-100071, | Tobacco industry |
| Dr Kishore Chaudhry, Asst. Director-General Indian Council of Medical Research (ICMR), New Delhi | Health |
| **Nine more members appointed on 13 September 1996 (Tobacco Institute of India & Sarkar, 1996)** |  |
| Dr. A.H. Rizavi, National Consultant, 2-A, Raj Narayan Road, Civil Lines, Delhi | Health |
| Shri P. Lakshman Swamy, 21/2, Paranacusapuram, Kodambakam, Madras | Health |
| Shri B. Madhu, Andhra Farmers Forum  House No .7/30/3 9 Math Road, Hotel Swapna, Near L.I.C., Rajahmundry, Andhra Pradesh | Farmers |
| Shri K. Appala Raju, President, The Godavari Farmers and Tobacco Growers Association, Polavaram, West Godavari District, Andhra Pradesh | Tobacco farmers |
| Shri K. Venkataratnam, President, The East Godavari District, Tobacco Growers and Farmers Association, Korukonda - (East Godavari District, Andhra Pradesh) | Tobacco farmers |
| Dr. P. Rao, C/o Indian National Trade Union Congress, West Godavari District, Unit, Eluru | Workers |
| Shri Tapas Ditta, General Secretary, All India Committee, United Trade Union Centre (Lenin Saran) and General Secretary, All Bengali Beedi Workers and Employees Federation, Calcutta | Workers |
| Shri H.B. Dave, General Secretary, Bharatiya Mazdoor Sangh, Ram Naresh Bhavan, Tilak-Gali, Fahar Ganj, New Delhi | Workers |
| Shri K.M. Shankar Prabhu, President, Karnataka Beedi, Industry Association, TVS Sadan, Kodumul Ranga Rao Road, Kodialbail, Mangalore Karnataka | Tobacco industry |

| **Table S9: Comparison COTPA 2003 and COTPA Amendment Bill 2020** | | |
| --- | --- | --- |
| **Key provision** | **COTPA 2003** | **Amendment proposed** |
| Preamble | No mention of WHO FCTC as it was not in force when COTPA, 2003 was enacted. | Mentions WHO FCTC. |
| Definitions | Section 3(a) Advertisement  Section 3(k) Production. | Redefines the two terms. |
| Smoke-free public places | Section 4 allowed designated smoking area/rooms. | Removes the exemption making all public places 100% smoke-free. |
| Advertising, promotion and sponsorship | Section 5 allowed concession on point of sale and in and on pack advertising. | TAPS ban on all mediums, explains terms like medium, promote and indirectly advertise. Removes concessions on PoS and in and on pack advertising. |
| Protection of minors | Section 6 prohibited sale to minors (<18 year) and within 100 yards of educational institutions. | No sale to anyone <21 years and within 100 meters of educational institutions. Increases penalty to up to seven years of imprisonment and fine up to INR 100,000/-. (INR 73= 1USD) |
| Pictorial health warnings | Section 7 provided pictorial health warnings on all products | New provision added requiring sale only in sealed, intact and original packaging i.e. no loose or single unit sale as minimum quantity per pack also to be prescribed. |
| Mention Tar and Nicotine on packs | Section 10 required mention of tar and nicotine on pack. Never invoked. | Deletes this provision. |
| Illicit trade and vendor licensing | No provision | Added section 10A to prohibit illicit trade and requirement of licensing for production, supply or distribution, import, sale, offer for sale of tobacco proudcts. |
| Penalties |  | 10-50 fold increase in penalties and offences punishable under sections 5, 6, 7 and 10A made cognizable |

**REFERENCES**

British American Tobacco Limited. (2020). *BAT-A Better Tomorrow*. British American Tobacco. Retrieved May 03 from <https://www.bat.com/group/sites/UK__9D9KCY.nsf/vwPagesWebLive/DOB4JJB8>

British American Tobacco Limited, & Opukah, S. (1996, 1996 February 02). *Voluntary Code* <https://www.industrydocuments.ucsf.edu/docs/slkb0207>

Business Standard. (2019). *Godfrey Phillips India jumps on Philip Morris, Altria merger talks*. Business Standard. Retrieved May 03 from <https://www.business-standard.com/article/news-cm/godfrey-phillips-india-jumps-on-philip-morris-altria-merger-talks-119082800717_1.html>

Godfrey Philips India Limited, & Poddar, R. (1996, January 08). *Letter from Rama Poddar to Amit Sarkar regarding voluntary code on cigarette advertising* <https://www.industrydocuments.ucsf.edu/docs/hmkb0207>

Saha, S. (2019). *Brickbats for BAT at ITC meet*. Telegraph India. Retrieved May 03 from <https://www.telegraphindia.com/business/brickbats-for-bat-at-itc-meet/cid/1694360>

Sarkar, A. (1994). *Shackles on the Mind or Freedom to Choose?* <https://www.industrydocuments.ucsf.edu/docs/kzvv0194>

Sarkar, A. (1995, September 11). *Expert Committee on Tobacco Use: Points for Discussion* <https://www.industrydocuments.ucsf.edu/docs/gkfk0208>

Shook Hardy & Bacon LLP. (1999, March 31). *Report on Recent International Developments Issue 1999 / 2 ; [Confidential Report on International Developments In Smoking and Health Legislation and Litigation Prepared by Philip Morris Outside Counsel Sent to Group Comprised of Philip Morris In-House Counsel and Philip Morris Outside Counsel for the Purpose of Providing Information and Legal Analysis Regarding Same]* <https://www.industrydocuments.ucsf.edu/docs/jgwb0008>

Tobacco Institute of India. (1992). *Our Responses to the Recommendations of the National Conference on Tobacco and Health* <https://www.industrydocuments.ucsf.edu/docs/txhh0214>

Tobacco Institute of India. (1996). *Proposed Voluntary Code for the Marketing of Tobacco Products* <https://www.industrydocuments.ucsf.edu/docs/qlkb0207>

Tobacco Institute of India, & Sarkar, A. (1996, 1996 September 19). *Expert Committee on Tobacco Formed by Union Ministry of Health* <https://www.industrydocuments.ucsf.edu/docs/lxvl0210>

Unknown. (1990, December). *Cigarette Advertising and Promotion Code* <https://www.industrydocuments.ucsf.edu/docs/tycp0208>

Unknown. (1992, April 02). *Tobacco & Health - Scenario* <https://www.industrydocuments.ucsf.edu/docs/hjhh0214>

Unknown. (1994a). *BAT Industries Future Business Environment 1995* <https://www.industrydocuments.ucsf.edu/docs/flpp0136>

Unknown. (1994b, February 25). *TOBACCO INSTITUTE OF INDIA VOLUNTARY CODE FOR THE MARKETING OF CIGARETTES IN INDIA* <https://www.industrydocuments.ucsf.edu/docs/xfvw0110>

Unknown. (1994c, April 18). *VOLUNTARY CODE FOR THE MARKETING OF TOBACCO PRODUCTS IN INDIA* <https://www.industrydocuments.ucsf.edu/docs/yzpd0110>

Unknown. (1995, November). *Committee on Subordinate Legislation: Twenty Second Report* <https://www.industrydocuments.ucsf.edu/docs/lmvl0210>
